# Levels of Valence

**DOI:** 10.3389/fpsyg.2013.00261

**Published:** 2013-05-13

**Authors:** Vera Shuman, David Sander, Klaus R. Scherer

**Affiliations:** ^1^Department of Economics, University of LausanneLausanne, Vaud, Switzerland; ^2^Swiss Center for Affective Sciences, University of GenevaGeneva, Geneva, Switzerland; ^3^Laboratory for the Study of Emotion Elicitation and Expression, Department of Psychology, University of GenevaGeneva, Geneva, Switzerland

**Keywords:** appraisal, common currency, emotion, pleasure, valence

## Abstract

The distinction between the positive and the negative is fundamental in our emotional life. In appraisal theories, in particular in the component process model of emotion (Scherer, 1984, 2010), qualitatively different types of valence are proposed based on appraisals of (un)pleasantness, goal obstructiveness/conduciveness, low or high power, self-(in)congruence, and moral badness/goodness. This multifaceted conceptualization of valence is highly compatible with the frequent observation of mixed feelings in real life. However, it seems to contradict the one-dimensional conceptualization of valence often encountered in psychological theories, and the notion of valence as a common currency used to explain choice behavior. Here, we propose a framework to integrate the seemingly disparate conceptualizations of multifaceted valence and one-dimensional valence by suggesting that valence should be conceived at different levels, micro and macro. Micro-valences correspond to qualitatively different types of evaluations, potentially resulting in mixed feelings, whereas one-dimensional macro-valence corresponds to an integrative “common currency” to compare alternatives for choices. We propose that conceptualizing levels of valence may focus research attention on the mechanisms that relate valence at one level (micro) to valence at another level (macro), leading to new hypotheses, and addressing various concerns that have been raised about the valence concept, such as the valence-emotion relation.

Valence is “one of the most important scientific concepts at the heart of emotion experience” (Charland, 2005, p. 83). An important question, then, is “what is valence?” The term *valence* was introduced by Lewin (1951) who used it in his field theory to refer to the forces that attract individuals to desirable objects and repel them from undesirable ones. The concept has since been considerably extended, including, but not limited to, the designation of emotions as positively or negatively valenced (for reviews, e.g., Solomon and Stone, 2002; Colombetti, 2005). There is strong agreement that valence, expressed with terms such as positive-negative, good-bad, or pleasure-displeasure, captures something essential about affect (Ortony et al., 1990; Solomon and Stone, 2002; Russell, 2003; Charland, 2005; Colombetti, 2005; Barrett, 2006; Frijda and Scherer, 2009). Researchers from various disciplines interested in emotions but also in motivation, learning, and decision making refer to the distinction between the positive and the negative with terms such as valence, pleasantness, utility, or liking/wanting.

The importance of the valence concept is also evident in appraisal theories, where pleasantness and goal conduciveness appraisal criteria have traditionally been seen as valence judgments (e.g., Frijda et al., 1989). Scherer (2010) recently suggested that other appraisal criteria in his component process model (CPM) are also valenced, such as power, self-congruence, and moral goodness. In the first part of the current paper, we describe these types of valence in more detail. The resulting multifaceted view of valence based on an appraisal framework is useful to predict and describe mixed feelings, and, in conjunction with other appraisals, to predict and describe emotions and action tendencies.

However, as will be discussed in the second part of the paper, the multifaceted conceptualization of different types of valence contrasts with the widely accepted view of valence as one-dimensional. For example, researchers note the need for a common currency in order to make choices (e.g., call mother or do laundry?), and valence has repeatedly been proposed to serve this purpose (e.g., McFarland and Sibly, 1975; McNamara and Houston, 1986; Cabanac, 1992; Shizgal and Conover, 1996; Montague and Berns, 2002; Peters et al., 2006; Pfister and Böhm, 2008). Although appraisal and one-dimensional valence “represent two major approaches to understand emotional experience in contemporary emotion research … [they] have largely lived side by side” (Kuppens et al., 2012, p. 1). Researchers have only very recently begun to examine the dynamic interplay between them in everyday experience (Kuppens et al., 2012).

In the third part of the paper, we propose a novel framework that may integrate the multifaceted with a one-dimensional view of valence by suggesting that valence should be conceived at different levels, micro and macro. Multifaceted micro-valences correspond to qualitatively different types of evaluations, potentially resulting in mixed feelings, whereas one-dimensional macro-valence corresponds to an integrative summary that informs choice. Conceptualizing levels of valence may focus research attention on the mechanisms that relate valence at one level (micro) to valence at another level (macro), leading to new hypotheses. We also discuss how our framework complements related models, such as the evaluative space model (Cacioppo and Berntson, 1994).

## Multifaceted Valence

Appraisal theory favors a multifaceted view of valence, proposing that emotions emerge as a consequence of events being appraised on multiple criteria. An appraisal consists of a subjective evaluation of (real, recalled, or fictitious) events or situations. Appraisals can be processed consciously or unconsciously by different cognitive systems (Leventhal and Scherer, 1987). The CPM (Scherer, 1984, 2001, 2009, 2010) proposes several appraisal criteria that are used to determine an event’s relevance and implications, the individual’s coping potential in the situation, and the normative significance of the event. Specifically, an individual can evaluate the relevance of an event by appraising its novelty, pleasantness, and goal relevance. Implications of an event are appraised with evaluations of causal attribution, outcome probability, discrepancy from expectations, goal/need conduciveness, and urgency. The individual’s coping potential depends on the general controllability of an event, the individual’s power to influence a situation, and the individual’s possibilities of adjusting to the situation. Information about the normative significance of an event is determined by comparisons to internal and external standards. The appraisals may be processed in a specific sequence (Scherer, 2001, 2009). For example, pleasantness is processed before goal conduciveness (e.g., Grandjean and Scherer, 2008). The assumption of recursive sequences of appraisals is particular to the CPM, but many of the proposed appraisal criteria are similarly described in other appraisal theories (Ellsworth and Scherer, 2003).

Of the different appraisals, pleasantness and goal conduciveness have traditionally been perceived as valenced (e.g., Frijda et al., 1989). Scherer (2010) recently proposed that the outcomes of additional appraisals can also be regarded as different types of valence. Here, we describe in much greater detail than has been done in previous CPM papers how the outcomes of five appraisals may be valenced: pleasantness/beauty, goal conduciveness, power, compatibility with the self (self-congruence), and compatibility with norms (moral goodness). We do not include all appraisal criteria, because our aim here is to illustrate the general point of multifaceted valence. Future research may extend the framework to include other appraisal criteria (e.g., novelty, certainty).

As a first type of valence, an evaluation can refer to pleasantness and beauty appraisal, related to the sensual or hedonic experience of a situation (Voss et al., 2003). Freud (1920) even argued that “our entire psychical activity is bent upon procuring pleasure and avoiding pain” (p. 311, italics removed). A common example for pleasurable experiences is eating good food, “one of life’s greatest pleasures” (Drewnowski, 1997, p. 243). In any situation, conflicting pleasantness appraisals are possible. For example, wearing high heels may feel painful but look pretty to a woman (Phelan, 2002). Thus, at any point in time, an experience can be both more or less unpleasant *and* more or less pleasant, in line with univariate notions of valence (e.g., Cacioppo and Berntson, 1994). Current homeostatic needs and basic physiological reactions may change temporary boundary settings for pleasantness experiences (e.g., Cabanac, 1979). For example, cool water may be unpleasant when one is cold and refreshing when one is hot (Cabanac, 1979). In contrast, an example for innate and less variable pleasantness is the pleasure derived from sweet tastes that shows high heritability and leads to similar reactions across species (Steiner et al., 2001; Keskitalo et al., 2007). A strong, genetically determined association of particular objects with (dis)pleasure is called intrinsic (un)pleasantness. Evolution may cause a certain level of invariability for the (un)pleasantness of things that are inherently harmful or beneficial for survival. In the case of sweetness preference, sugar preference may have evolved because sugar is an easily detectable, though rough, indicator for eatable foods (Ramirez, 1990). Although intrinsic pleasantness may be rooted in ultimate evolutionary benefits, it can be differentiated from proximal goal conduciveness.

Goal conduciveness is a second, qualitatively different, type of valence. The determinant of goal conduciveness is the functionality or efficiency of a situation to satisfy needs, achieve goals, or confirm values. The role of homeostatic needs differs for goal conduciveness and pleasantness. The degree of goal conduciveness is directly determined by the degree of need satisfaction or goal attainment. In contrast, needs may set thresholds for pleasantness, but cannot predict pleasantness beyond those thresholds. An event can satisfy goal attainment directly or by facilitating further goal-directed behavior. For example, food may be more or less conducive to health or appearance goals, and thus be at the same time goal conduciveness in one regard *and* goal obstructive in another regard (e.g., Lindeman and Stark, 1999; Cramer and Antonides, 2011), consistent with univariate notions valence (e.g., Cacioppo and Berntson, 1994). Pleasure and goal conduciveness often co-occur. For example, in natural reward learning, pleasure experiences may trigger the learning of stimulus- or behavior-reward associations, followed by the attribution of goal conduciveness to the newly associated instrumental stimulus or behavior (e.g., Berridge and Valenstein, 1991). However, empirical research shows that pleasure is independent of goal conduciveness. The sensual enjoyment of an object and the goal striving toward an object rely on different brain mechanisms (e.g., Berridge, 2003). Also, hedonic and utilitarian dimensions can be empirically distinguished as independent variables in consumer attitudes (e.g., Voss et al., 2003). The dissociation between the positive valence associated with each goal achievement and pleasantness can lead to the paradoxical effect that individuals strive harder to acquire consumer goods they like less (e.g., Litt et al., 2010). Finally, the distinction of pleasantness and goal conduciveness is also evident with regard to sugar preference. Evolved sugar preference can be attributed to pleasantness rather than goal conduciveness appraisals, because sweetness can elicit positive affect, even if the sweet drink is considered too sweet for consumption (Booth et al., 2010). Moreover, sugar is not a particularly sensitive indicator for the nutritive value of a food (Ramirez, 1990). In addition to the evolved tendency of being evaluated positively, sugar may, particularly in more developed countries, be appraised as goal obstructive to health and appearance goals.

Third, the outcome of a power appraisal is valenced. Power appraisals refer to the ability of an individual to have influence by one’s own actions or by mobilizing others (Scherer, 1984, 2009). High power is associated with positive affect and low power with negative affect (e.g., Keltner et al., 2003). Because of the importance of personal influence, the role of resources differs for power and goal conduciveness. Power is tightly linked to personal control over resources. In contrast, goals may also be achieved with resources outside one’s personal control. For example, my goal to live in personal safety is to a large extent achieved by means that are outside my personal control, for example, by police and laws. In the CPM, power appraisals only matter when an event is appraised as generally controllable by humans, animals, or human artifacts (Scherer, 1984, 2009). For example, one may perceive running a marathon after 4 months of training as generally feasible. Once the event is appraised as controllable, personal failure in running a marathon may result in feeling powerless. The importance of controllability appraisals for power suggests a coping strategy when feeling powerless. Once the event is appraised as uncontrollable (e.g., nobody could run a marathon after only 4 months of training), feelings of power are no longer threatened. A related concept to power appraisals is self-efficacy beliefs (Bandura, 1977). These beliefs about personal ability to influence a particular situation may initiate and sustain coping behavior, increasing the likelihood of experiencing positively valenced feelings of power.

The fourth type of valence is based on appraising an event as congruent with one’s self-concept. Individuals generally like congruence, balance, and harmony, and these can also be applied to the self. According to Higgins (1987), there are several self-domains. The “actual self” refers to how a person perceives him- or herself to actually be, the “ought self” to what the self should be like based on norms or duties, and the “ideal self” to what the person aspires to be. These domains can be based on one’s own or another’s standpoint. Previous CPM work (Scherer, 1984, 2009) emphasized the distinction between internal and external norms, but here we refer to the actual self and the ought/ideal self to characterize self-congruence and moral goodness, respectively. For example, food choices may reflect one’s identity and result in feeling self-congruent (Lindeman and Stark, 1999). The self includes traits, internalized roles, and social identities. Self-congruence is therefore applicable to conceptualizations of the independent or interdependent self (Markus and Kitayama, 1991). The importance of self-congruence is captured in multiple psychological concepts, such as cognitive dissonance (Festinger and Carlsmith, 1959), self-affirmation (Steele and Liu, 1983), and self-verification (Swann and Read, 1981). Trait self-congruence is positively associated with self-esteem, life satisfaction, and positive affect (Goldman and Kernis, 2002). In contrast, conflicting internalized roles may result in feelings of incongruence. For example, individuals may have conflicting gender and work identities (Sacharin et al., 2009); wearing a purse may be in accordance with a female field engineer’s gender identity *and* at the same time not be in accordance with her work identity (Miller, 2004).

A fifth type of valence, based on appraisal of an event as being in accord with one’s ought and ideal self, is moral goodness. Duties and ideals are defined in a social context and can be more or less internalized. For example, there are multiple ideals and duties for members of a religion. Even food may be the object of moral goodness: it may be offered to the gods to assure their goodwill (Appadurai, 1981). Other duties and ideals extend the social group to all humanity (e.g., categorical imperative). Typically, moral goodness ensures group cohesion and helps to satisfy the need to belong (Baumeister and Leary, 1995). However, ideals may also be construed in opposition to a common moral code (e.g., anarchists). In this paper, we do not distinguish sharply between moral and conventional rules. In the moral domain (e.g., “you shall not kill”), evaluations may occur in an all-or-nothing fashion: good or bad, right or wrong; in the domain of conventions (what dress to wear to a wedding), appraisal results may be more graded. For both domains, feelings are essential in learning and maintaining how to “be good.” At any moment in time, an experience can contain both morally good and bad elements, consistent with a univariate notion of valence (e.g., Cacioppo and Berntson, 1994). For example, images of charity work contain morally bad elements (e.g., injustice reflected in poverty) and morally good elements (helping behavior). Research on self-conscious affect suggests that the appraisals of self-congruency and moral goodness may often occur slower than other appraisals, such as pleasantness, and be more deliberate; however, they can also become internalized resulting in fast and unconscious processing (e.g., Giner-Sorolla, 1999).

The multifaceted view on valence of the CPM is highly compatible with the notion of mixed feelings. The CPM suggests three different routes to mixed positive and negative affect. First, there may be conflicts within an appraisal, for example when an image of charity work contains features that lead to appraisals of moral goodness and of moral badness. Second, different appraisals may conflict. For example, pleasure and goal conduciveness can be in conflict when sugary foods taste good to person on a diet, resulting in feeling good and bad at the same time. Third, conflicts can arise between systems of processing of an appraisal. For example, the association between goal conduciveness and a stimulus can be implicitly learned (schematic level) or can be learned from rules (conceptual level), and these processing systems can conflict (Leventhal and Scherer, 1987; Van Reekum and Scherer, 1997; compare also Rangel et al., 2008). Indeed, evidence is accumulating that individuals can simultaneously feel positive and negative affect (e.g., Diener and Iran-Nejad, 1986; Scherer and Ceschi, 1997; Larsen et al., 2001; Schimmack, 2001, 2005; Scherer et al., 2004; Oceja and Carrera, 2009; Larsen and McGraw, 2011).

Furthermore, the CPM suggests how multifaceted valence may be related to specific behavioral tendencies based on the multicomponential view of emotions. The components are appraisals, subjective feelings, physiological changes, motor expressions, and action tendencies. Appraisals are regarded as driving changes in the other emotion components leading to full-blown emotions when the different components are synchronized (Scherer, 2009). Appraisal theory specifies appraisal profiles for different emotions (e.g., Smith and Ellsworth, 1985; Roseman et al., 1990; Smith et al., 1993; Scherer, 1997; Kuppens et al., 2007; Siemer et al., 2007; Tong et al., 2009). For example, the combination of unpleasantness and moral badness of the situation, in conjunction with other appraisals, has been associated with anger, and the combination of unpleasantness of the situation and low power of the individual with sadness (e.g., Scherer, 1997). No one appraisal may be necessary or sufficient for an emotion, but the occurrence of an emotion may require a subset of the proposed appraisals in appraisal theory (Kuppens et al., 2003; Parkinson and Roper, 2009). The integration of appraisals may not only depend on which appraisals are combined, but also on the nature of the appraised situation. Specifically, Ortony et al. (1990) describe how particular appraisals regarding events, objects, or actions and concerning the self or others are associated with emotions. Even more elaborate integration functions for the appraisal-emotion relation have recently been proposed. On the basis of Anderson’s (1989) model of integration functions Scherer (2004) suggests that the type of integration rule for the combinations of appraisal criteria may depend on an individual’s current goals. For example, coping ability is of less relevance when things are going according to plan. In Anderson’s approach, this would be modeled by a configuration rule in which the importance of one criterion depends on the level of another. Non-linear dynamic system analysis is a more appropriate framework for emotion modeling of such integration functions than is the classic assumption of linear functions (Scherer, 2000).

Commonly, appraisals are linked to action tendencies via emotions. For example, sadness is associated with the action tendency of helplessness and anger with antagonistic behavior (Frijda et al., 1989). Studying more directly the relations between emotion components, appraisal theorists showed that appraisals are associated with specific action tendencies (e.g., Frijda et al., 1989). Efferent effects occur in the autonomic nervous system (e.g., in the form of cardiovascular and respiratory changes) and in the somatic nervous system (in the form of motor expression in face, voice, and body; e.g., Van Reekum et al., 2004).

To a limited extent, it is also possible to predict choice from appraisals given the existing theoretical development of appraisal theory. For example, appraisals of high and low control may inform the choice between a high- and low-risk option (Lerner and Keltner, 2000). However, without further extending appraisal theory, it is not possible to predict choice more generally. For example, how does a person choose between a pleasant but goal-obstructive option (e.g., reading Facebook at work) and one that is unpleasant but goal conducive (e.g., proofreading an article)? Appraisal theory was not developed to answer these questions, or to predict choice behavior more generally, but here we suggest that it can be further developed to that end.

To summarize, four strengths of a multifaceted valence concept based on appraisal theory have been discussed (there may be more): (a) qualitative differences between events can be described (e.g., pleasant versus goal conducive events); (b) appraisal theory can explain mixed feelings; (c) mechanisms are proposed for how valences combine to form emotions; and (d) combinations of valences can be used to predict specific action tendencies. Appraisal theory requires further development before choice across situations can be explained. We propose in this paper that an integration of the multifaceted view of valence with a one-dimensional view of valence may be useful to solve this problem. In the following, we discuss the advantages and ubiquity of a one-dimensional valence concept. We also briefly review the problems associated with one-dimensional valence.

## One-Dimensional Valence

The one-dimensional valence concept may be the key to understanding how behavior is prioritized (e.g., Cabanac, 1992). There is a logical need for the integration of complex affective experiences into a common currency to compare, rank, and choose between options (e.g., McFarland and Sibly, 1975; McNamara and Houston, 1986; Cabanac, 1992; Shizgal and Conover, 1996; Montague and Berns, 2002; Pfister and Böhm, 2008). It has repeatedly been proposed that valence may function as that common currency (e.g., Cabanac, 1992; Russell, 2003; Barrett, 2006; Peters et al., 2006). Valence is here closely related to the notion of utility, which similarly captures total satisfaction with a good or service and influences preferences (Kahneman and Tversky, 1979; Shizgal and Conover, 1996; Kahneman et al., 1997; Montague and Berns, 2002; Russell, 2003).

Cabanac and colleagues showed in multiple experiments that valence may function as a common currency (for a review, see Cabanac, 1992). For example, participants walked on a treadmill and rated the discomfort in their chest and in their legs. In a different session, participants could adjust the speed of the treadmill at various slopes or vice versa. Participants’ choices reflected the algebraic sum of their previous discomfort ratings (Cabanac, 1985). In a different study, the duration of enduring a painful position could be modeled as the algebraic sum of subjective pain and money reward (Cabanac, 1986). In another study, after participants rated the pleasantness of sandwiches, they could then choose what to have for lunch by paying money for sandwiches they liked or by receiving money for sandwiches they disliked in various sessions with different payment structures. The chosen sandwiches reflected the area of optimal compromise between pleasure and cost (Cabanac, 1995). Other research, too, supports the notion of a common currency. For example, individuals consider the pleasantness of an anticipated emotion as well as its usefulness, and may even choose situations that arouse unpleasant emotions if they believe that these emotions are goal conducive (Tamir, 2009).

Given its importance for choice, it is not surprising that valence is evident early in ontogenetic development. For example, it has been suggested that signs of distress can already be differentiated from general arousal in 3-week-old infants (Bridges, 1932). Underlying the expression of negative affect is presumably the ability to appraise events as positive or negative. Furthermore, sweet and bitter tastes arouse homologous behavioral patterns in newborns and in non-human primates (Steiner et al., 2001). As indicated by studies on (un)pleasant and goal conducive (obstructive) events, many of the neural correlates for valence seem to be shared with other mammalian species (e.g., Berridge and Kringelbach, 2008). This suggests that positive and negative valence is an evolutionary old distinction.

Furthermore, valence emerges repeatedly as the dimension that explains most variance in the classifications of affective words, facial and vocal expressions, and affective states aroused by various stimuli across language and age groups; other dimensions that characterize the affective space are arousal, dominance, and novelty (e.g., Fontaine et al., 2007, in press). Across language and age groups and in patients and non-patients, analyses of emotion words used to describe affective states repeatedly show a valence dimension as the underlying organizational structure with the greatest explanatory power (e.g., Block, 1957; Bush, 1973; Russell and Mehrabian, 1977; Russell, 1978, 1980; Russell and Ridgeway, 1983; Russell et al., 1989; Reisenzein, 1994; Feldman, 1995; Kring et al., 2003). In addition, facial expressions can be structured along a valence dimension (e.g., Schlosberg, 1954; Abelson and Sermat, 1962; Russell and Bullock, 1985; Russell et al., 1989). Valence also explains the most variance regarding the underlying structure of subjective judgments of affective vocal stimuli (e.g., Green and Cliff, 1975). Furthermore, self-reported affective states in everyday life and in response to hypothetical scenarios, affective pictures, and colors can be organized along a valence dimension (e.g., Russell and Mehrabian, 1977; Russell and Steiger, 1982; Bradley and Lang, 1994; Valdez and Mehrabian, 1994; Feldman, 1995; Barrett, 1996; Barrett and Russell, 1998; Yik et al., 1999). Similarly, when taking multiple components of the affective experience jointly into account, the valence dimension captures most of the variance in the data, as shown in cross-cultural studies on the underlying structure of appraisals, psychophysiological changes, motor expressions, action tendencies, subjective experiences, and emotion regulation (Fontaine et al., 2007, in press). The reliability with which a valence dimension appears in these studies suggests that valence is always present in human affective life (Russell, 2003).

A strong case can be made for the necessity and existence of one-dimensional valence. Not surprisingly, one-dimensional valence plays a central role in current emotion theories, such as theories of core affect and the psychological construction of emotion in which valence constitutes, together with arousal, core affect (Russell, 2003; Barrett, 2006).

However, there are also several problems with a one-dimensional valence concept. First, the role of a single valence dimension for behavior prediction is limited. Although positive affect has been associated with a generative behavioral orientation (exploring, achieving positive outcomes, risk taking, little loss aversion) and negative affect with a defensive behavioral orientation (avoiding negative outcomes; Seo et al., 2010), other researchers suggest that approach and withdrawal may be related only to particular positive and negative emotions (Davidson, 1994). Importantly, emotions that cannot be distinguished based on their valence, such as fear and anger (Scherer, 2005), may influence cognition and behavior in different ways (e.g., Zeelenberg and Pieters, 2006). For example, anger reduces the perception of risk, but fear increases it (Lerner and Keltner, 2000). Also, anger has been associated with approach behavior, but fear with avoidance behavior (e.g., Carver and Harmon-Jones, 2009).

Second, a one-dimensional valence concept is at odds with research findings on mixed feelings. Evidence is accumulating that individuals feel mixed emotions at the same time (e.g., Diener and Iran-Nejad, 1986; Scherer and Ceschi, 1997; Larsen et al., 2001; Schimmack, 2001, 2005; Scherer et al., 2004; Oceja and Carrera, 2009). Given the limitations in the temporal resolution of the measurement of affect, the existence of true mixed emotions has been questioned, with some arguing that positive and negative emotions vacillate rather than co-occur (e.g., Barrett and Bliss-Moreau, 2009). However, increasingly sophisticated measurement techniques suggest that mixed emotions do exist (e.g., Larsen and McGraw, 2011).

Third, the assignment of emotions to valence is, upon closer inspection, ambiguous (Solomon and Stone, 2002; Charland, 2005; Colombetti, 2005; Zeelenberg and Pieters, 2006; Pfister and Böhm, 2008; Frijda, 2009). For example, theoretically a situation causing anger may be experienced as negative, but the arousal associated with being angry experienced as positive (e.g., Pfister and Böhm, 2008). The theoretically derived ambiguity of emotions like anger contrasts with the common empirical finding that anger is a negative emotion from studies on the structure of affect reviewed above as well as from studies with a direct assessments of the perceived valence of anger (e.g., Bänziger et al., 2005). This poses an unresolved puzzle that will be discussed in a later section of this paper.

Fourth, using a one-dimensional valence concept to characterize an emotion (e.g., fear is negative) leads to conflation in the causes, feelings, consequences, and other aspects associated with the emotion as negative (Colombetti, 2005), which may in many cases not be justified (e.g., fear has the positive consequence of avoiding danger; Colombetti, 2005). Similarly, shame may feel negatively, but the effect it has on encouraging normatively appropriate behavior can be regarded as a positive consequence. The problem is that the descriptive finding from one domain (e.g., fear/shame feels bad) may lead to prescriptive judgments (fear/shame is a negative emotion) and unjustified implications (fear/shame is to be avoided).

Finally, using a one-dimensional valence concept ranging from pleasant to unpleasant may foster dichotomous thinking (good versus bad, positive versus negative), implying that “less positive” means “more negative,” which is not necessarily true (Colombetti, 2005). For example, positive or negative attitudes have distinguishable causes and consequences (e.g., Cacioppo and Berntson, 1994).

To summarize, we have discussed three major strengths of the one-dimensional valence concept (we do not rule out that there may be more strengths): (a) one-dimensional valence corresponds to the empirically emerging structure of affective life; (b) one-dimensional valence is necessary for choice; and (c) the distinction of positive and negative may be evolutionary old. The weaknesses of a one-dimensional valence concept are that (a) it is difficult to predict action tendencies beyond a general generative or defensive orientation from valence; (b) mixed felt affect cannot be explained; (c) associating emotions with valence is ambiguous; (d) the concept may lead to unjustified conflations; and (e) the concept may lead to dichotomous thinking. Given these problems, some researchers have even come to the conclusion that “the analysis of emotions in terms of ‘valence’ … is an idea that we should abandon and leave behind” (Solomon and Stone, 2002, pp. 431–432; Zeelenberg and Pieters, 2006).

## A Framework for Levels of Valence

How can the idea that qualitatively different types of valence exist be reconciled with the proposition that valence can serve as a common currency for choice? Which of the perspectives should be preferred, given that they both have advantages and disadvantages? Instead of believing that only either qualitatively different types of valence or a “common currency” valence exists, we suggest that both views can be reconciled by assuming that valence can exist at two levels. We propose a new theoretical framework that bridges the opposing notions of valence. We suggest that valence is located, first, at the level of individual appraisal outcomes (*micro-valence*) and second, at the level of an integration of various inputs, which may or may not include micro-valences, into a *macro-valence*. Importantly, in addition to suggesting *that* micro-valences *and* macro-valence occur (e.g., compare Cabanac, 1992), we propose specific mechanisms for the nature of the relation between micro- and macro-valence.

**Figure 1:**
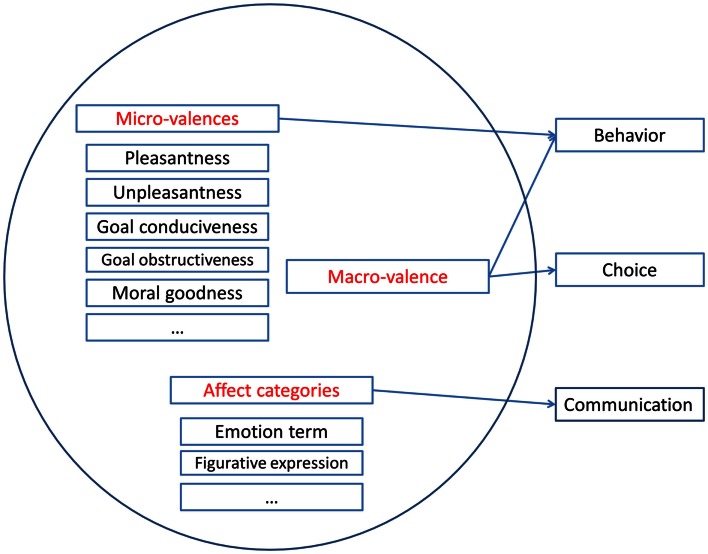
**The different functions of micro-valences, macro-valence, and affect categories**. Various micro-valences (individual evaluations, positive and negative dimensions), macro-valence (an overall affect ranging from negative to positive), and affect categorization co-occur in time. Micro-valences and macro-valence influence behavior, macro-valence is necessary for choice, and affect categories are used in communication. Displayed are the appraisal-based micro-valences discussed in the paper, but other micro-valences are possible. The figure displays some components of emotions (some appraisals in micro-valences, action tendencies in behavior, and expression in communication), but not all (e.g., other appraisals, physiological changes, and subjective feeling, e.g., Scherer, 2009).

Figure 1 shows a schematic overview of the proposed theoretical framework. It entails multifaceted micro-valences, one-dimensional macro-valence, and affect categories. When interacting with the environment (for example, when browsing Facebook at work), an individual (e.g., Alex) evaluates a situation by using different appraisal criteria. For example, Alex may be able to evaluate the pleasantness of the situation, how goal conducive it is, how much control he or she has, how well the situation agrees with the self-concept, and how normatively appropriate the situation is. Although Alex may have a large repertoire of appraisal criteria at his or her disposal, some criteria may be more salient, or in the foreground, than others. For example, Alex may think mostly about the pleasantness, how well the situation serves the goals of maintaining a social network and getting work done, and the event’s normative significance. Alex may then come to a number of appraisal results, that is, micro-valences (e.g., the situation is fun, good for the goal of maintaining a social network but bad for getting work done, and morally bad). Thus, the overall experience may be multifaceted and lead to conflicting feelings.

It may be important to compare the situation to a different situation, for example, when deciding among behavioral options for the next day (Facebook? work?). For choice among behavioral alternatives (i.e., in order to make a decision), even though there may be negative and positive aspects of each alternative, there needs to be a net outcome of bipolar macro-valence (e.g., feeling “good” about reading Facebook and “somewhat bad” about doing work, and thus choosing to read Facebook).

Furthermore, Alex may want to express how he or she feels when talking to a friend. Humans semantically categorize their affective states and communicate them to others (e.g., Rimé, 2009). The expression can take multiple socially agreed upon forms, such as facial expressions, gestures, postures, tones of voice, words, and music. With words, combinations of micro-valences can be expressed in prototypical emotion terms (e.g., anger, happiness) or mixed emotion terms (e.g., nostalgia, Schadenfreude), figurative expressions, or more lengthy explanations (Scherer and Ceschi, 1997; Scherer et al., 2004). For example, Alex may use emotion terms and say he or she feels happy when reading Facebook, but bored when doing work. These emotion terms capture valenced and non-valenced appraisals and additional emotion components, such as physiological changes and action tendencies. Rather than using emotion terms, Alex may use a metaphor and say he or she feels blue when doing work. Another alternative is to say that he or she feels very good about reading Facebook and somewhat bad about working, or, even more succinctly, that he or she feels good overall about reading Facebook at work. For communicating one’s state, positive and negative may be construed as bipolar or as bivariate. The categories used to express one’s feelings to others may be highly dependent on current cultural norms and rather fuzzy. When communicating an affective experience, some information may get lost. Additionally, meanings that were not part of the affective experience may be added by the perceiver. The less-than-perfect match between experience, expression, and perception is a general problem of communication that affects all forms of communication.

**Figure 2:**
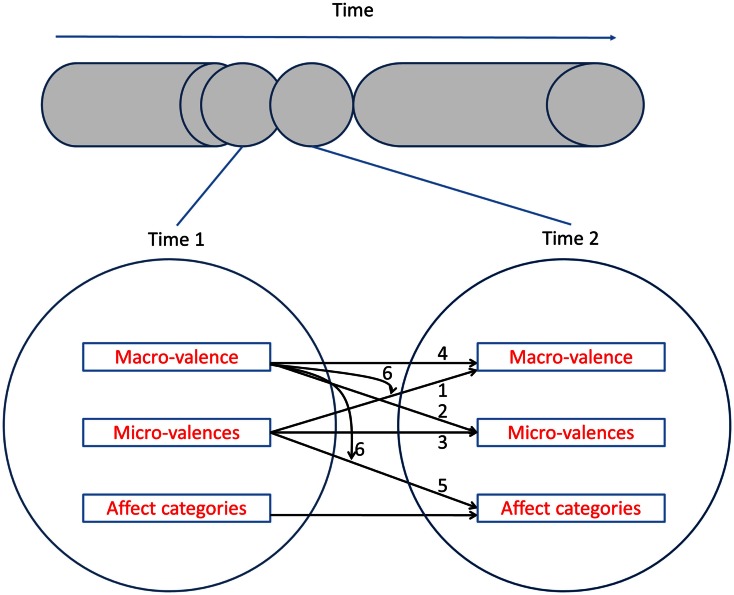
**Macro-valence, micro-valence, and affect categories over time**. Macro-valence, micro-valence, and affect categories co-occur in time, and influence subsequent macro-valence, micro-valence, and affect categories as indicated by the arrows. The numbers (1–6) correspond to the Sections “From Micro-Valences at Time *t* to Macro-Valence at Time *t* + 1,” “From Macro-Valence at Time *t* to Micro-Valence at Time *t* + 1,” “From Micro-Valence at Time *t* to Micro-Valence at Time *t* + 1,” “From Macro-Valence at Time *t* to Macro-Valence at Time *t* + 1,” From Micro-Valence at Time t to Emotions at Time *t* + 1,” and “Macro-Valence at Time *t* Moderates the Paths from Micro-Valence at Time *t* to Emotions and Macro-Valence at Time *t* + 1” in the paper where the paths are discussed.

The above example may suggest that micro-valences occur before macro-valence, but as illustrated in Figure 2 and described further below in the current framework micro-valences, macro-valence, and emotions are modeled with no strict primacy in time of one component over the others. Instead, multiple possible pathways across time are specified, including micro-valences that precede macro-valence and macro-valence that precedes the integration of micro-valences to emotions. The framework is therefore highly compatible with the notion that affective experiences are not “static phases, but … dynamic phenomena of which the components continuously change and follow each other across time (Kuppens et al., 2012, p. 7). Researchers can aim at describing, explaining, and predicting one part of this dynamic (e.g., how macro-valence follows from an integration of previous micro-valences) or another (e.g., how macro-valences influence subsequent micro-valences).

We suggest that macro-valence is always present, need not have an object, and may have multiple causes ranging from psychological to physiological. Although macro-valence is justified by its utility in a choice situation and then obviously has an object, we suggest that macro-valence is also present in non-choice situations and may be without a particular object, such as in the case of a mood that is typically defined as having no object. With regard to these features, macro-valence is similar to “core affect” valence in psychological constructionist models of emotion (Russell, 2003; Barrett, 2006). In contrast, micro-valences have an object, not all appraisals may be available at birth (e.g., moral goodness), and they may differ in salience; as a result, not *all* micro-valences may be present at every moment in time. However, the subset comprising relevance appraisals, including pleasantness appraisals, is likely to be continuously processed. Processing of micro- and macro-valences and affect categories may occur consciously or unconsciously (e.g., Van Reekum and Scherer, 1997).

Furthermore, although some researchers have described valence as “hot” and contrasted it with “cold” judgments, such as appraisals (Russell, 2003), we do not make this distinction. Valence is not restricted to emotions, but is also relevant for attitudes and preferences (Cacioppo et al., 1999). Similar processes may relate multifaceted micro-valences with one-dimensional macro-valence in “hot” and “cold” judgments.

Most important, we propose that the relations between micro- and macro-valence and affect categories deserve more attention in the research agenda of affective scientists. We discuss existing evidence and propose new hypotheses for the paths displayed in Figure 2, and also justify the absence of certain paths. We then discuss the implications of the framework for mixed feelings and choice situations.

### From micro-valences at time *t* to macro-valence at time *t* + 1

In a recent study on affective experience in daily life, Kuppens et al. (2012) examined whether appraisals at time *t* predict valence at time *t* + 1. They found that motivational congruence, coping potential, and future expectancy appraisals predicted more general feelings of valence [operationalized as feeling (un)pleasant]. Kuppens et al. did not use the CPM appraisal checks but were oriented by the framework of Smith et al. (1993); nonetheless, their results are highly relevant for the current paper, because their research demonstrates, for the first time, the influence of appraisals on valence in everyday experiences. Furthermore, the authors found large variance across persons in the appraisal-valence relation, suggesting that the influence of appraisals on valence differs across individuals.

To better understand how such individual differences may come about, and to predict in more detail how micro-valences may influence macro-valence, multiple hypotheses for the underlying integration function can be derived in particular from behavioral economic models and animal behavior models on choice. These models describe how attributes are integrated to inform choice normatively (how is the objectively best choice achieved?) or by observing decision making with hypothetical scenarios. Additionally, the processes that underlie inferences in decision making may be similar to the processes that underlie preferences (e.g., Gigerenzer and Gaissmaier, 2011). Taken together, this research suggests several processes for the integration of micro-valences to macro-valence. A common distinction is between processes in which all available information is taken into account versus those in which some information is ignored.

First, micro-valences may be integrated to macro-valence by a weighted sum (e.g., Keeney and Raiffa, 1976; Hammond et al., 1998; Mellers, 2000). For each alternative (e.g., a situation or an object), the macro-valence across its appraisals is computed before the alternative is compared to other alternatives. Furthermore, with dynamic models (e.g., McNamara and Houston, 1986), the integration of evaluations is dependent on the previous state of the system. It remains to be tested whether the integration of micro-valences at time *t* to macro-valence at time *t* + 1 depends on the state of the micro-valences at time *t* − 1. Without this effect, one’s overall feeling, for example, when taking a warm shower would be influenced only by the current temperature of the water. With a dynamic model, the previous water temperature would also be important.

Second, instead of using a decision function that requires the extensive processing of all micro-valences in a situation, choices can be simplified by (a) taking all micro-valences into account with equal weights (so-called tallying or Dawes’ rule; e.g., Gigerenzer and Gaissmaier, 2011), (b) by focusing on a limited number of micro-valences (e.g., in a lexicographic or a elimination-by-aspect strategy, see below), or (c) by only taking one micro-valence into account (so-called one-clever-cue heuristic; Gigerenzer and Gaissmaier, 2011). More specifically, a lexicographic decision rule refers to the strategy where alternatives are successively selected on the basis of the best option regarding the most important micro-valence(s). With an elimination-by-aspect strategy (Tversky, 1972), alternatives are successively eliminated that do not meet a minimum level for the important micro-valence(s). For example, when deciding among several options, an individual may follow an elimination-by-aspect strategy and find moral goodness more important than pleasantness. She may choose to eliminate all options that do not meet a minimum level of moral goodness followed by eliminating all remaining options that do not meet a minimum level of pleasantness. The individual may subsequently use a lexicographic decision rule and select all options that have the highest level of goal conduciveness followed by choosing the option with the highest level of self-congruency. In both strategies, no trade-offs between micro-valences are required (Luce et al., 1997). In other words, the individual would not need to compare how much moral goodness corresponds to how much pleasantness.

What determines which process individuals use to integrate micro-valences to macro-valence? Firstly, whether more or less information is processed can depend on the trade-off between accuracy and effort goals (e.g., Payne et al., 1996). Research on positive monetary gambles shows that an accuracy goal may initiate a processing-intense function, resulting in a preference for the option with the highest expected value, whereas a minimizing-effort goal leads to a less processing-intense, lexicographic decision rule (Payne et al., 1996). The latter may be advantageous, for example, when multitasking (Bless et al., 1996). Secondly, appraisals of uncertainty may be relevant in determining how much processing effort is expended (Tiedens and Linton, 2001). Emotions associated with high certainty (happiness and anger; e.g., Ellsworth and Scherer, 2003) increase stereotypical information processing that relies on heuristics and scripts (Forgas, 1992; Bodenhausen et al., 1994; Forgas and Fiedler, 1996; Tiedens, 2001). In contrast, low certainty affect is associated with more deliberate information processing (e.g., Forgas and Fiedler, 1996). Thirdly, the information integration process is often based on an ecological rationality, such that individuals tend to use the process that performs best in a particular type of environment (Gigerenzer and Gaissmaier, 2011). In order to apply this proposition to macro-valence, one has to define “best performance.” We suggest that a macro-valence is “better” when it is reliable (e.g., it does not change its value over time) and valid (e.g., it corresponds to overt behavior, self-report, etc.). In many cases, this may be a lower effort process (Gigerenzer and Gaissmaier, 2011), though not necessarily an unconscious process (e.g., Acker, 2008).

As recent reviews of dual-process theories show, one can distinguish processes that rely on working memory from those that do not (e.g., Evans, 2008). Although the two classes of processes are often described as, on the one hand, unconscious, fast, and automatic, and, on the other hand, conscious, slow, and deliberative, these features do not necessarily coincide (Moors and De Houwer, 2006; Evans, 2008; Gigerenzer and Gaissmaier, 2011). For example, lexicographic strategies are common in conscious and unconscious processing (Huizenga et al., 2012). As a result, the question of how many micro-valences are integrated to macro-valence, and how conscious this process occurs are independent issues. We will return to the issue of consciousness in the Section “Implications for Mixed Feelings and Choice.”

As described earlier, a lexicographic and an elimination-by-aspect strategy require the identification of the most important micro-valence. This also plays a role for more extensive integration functions in which micro-valences may be weighted differently on the basis of their importance (e.g., Keeney and Raiffa, 1976). Some research suggests that specific micro-valences may generally be more important than others. For example, moral goodness may generally dominate other micro-valences as suggested by a study in which choices were facilitated when one of the alternatives was morally better than the other (Hanselmann and Tanner, 2008). However, this study with hypothetical scenarios did not systematically compare the importance of various micro-valences, some of which, such as pleasantness, may be more important in non-hypothetical situations. The prediction that moral goodness trumps other micro-valences in its influence on macro-valence therefore requires further research.

Other research suggests that there are systematic individual differences in the importance of micro-valences. For example, individuals high in sensation seeking (Zuckerman et al., 1964) or who value hedonism (Schwartz and Bardi, 2001) may regard pleasantness as more important than others, as indicated by research on sensation seeking and movie enjoyment (Eliashberg and Sawhney, 1994). Systematic “appraisal biases” (Scherer and Brosch, 2009) in cultures and individuals may reflect differences in the salience or in the importance of micro-valences. One could therefore predict that individual and cultural differences are systematically related to the influence of micro-valences on macro-valence.

Finally, negative and positive evaluations are weighted differently. Research on attitudes, impression formation, decision making, and behavior shows that negative evaluations are weighted more heavily than positive evaluations (negativity bias; e.g., Miller, 1959; Kahneman and Tversky, 1979; Cacioppo et al., 1999). Furthermore, low levels of positive stimulus input may lead to more positive evaluations than low levels of negative stimulus input lead to negative evaluations (positivity offset; Cacioppo et al., 1999). Papers on the evaluative space model review in detail the distinguishable causes, neural and physiological correlates, and consequences of positive and negative evaluations (Cacioppo and Berntson, 1994; Cacioppo et al., 1999). Future research is needed to systematically examine whether negativity bias and positivity offset occurs for all micro-valences.

The evaluative space model furthermore suggests that “the value of separate and multifaceted inputs (is translated) onto common evaluative (positive, negative) metrics” (Ito and Cacioppo, 2005, pp. 1–2) before “physical limitations constrain behavioral expressions and incline behavioral predispositions toward a bipolar (good-bad, approach-withdraw) organization” (Cacioppo et al., 1999, abstract). In other words, all positive and all negative micro-valences may be integrated separately on positive and negative dimensions before they combine to macro-valence. This hypothesis remains to be tested.

The integration of micro-valences to macro-valence may not be particularly stable. For example, the integration of strongly positive and negative micro-valences may be unstable because it is experienced as a conflict, and individuals who appraise this conflict, e.g., when in a choice situation that cannot be postponed, are likely to regulate their state to reduce the conflict (Cacioppo and Berntson, 1994; van Harreveld et al., 2009). Also, integration may be difficult in situations involving the trade-off of various moral goodness evaluations (Hanselmann and Tanner, 2008) or with particularly complex situations, such as job choices (e.g., Luce et al., 1997; Hammond et al., 1998). In the example of a job choice situation, an impasse may occur in evaluations of the goal conduciveness associated with the salary of job A and the self-congruency associated with the departmental fit of job B. Stability, in this example, may only be achieved over time when new appraisals become salient (e.g., by adding the pleasantness of the weather at location A into the decision making), when particular appraisal results change (e.g., the salary difference between A and B will not substantially improve one’s standard of living), or when the integration parameters change (e.g., salary is not as important as the departmental fit). These mechanisms may be used to reduce cognitive dissonance (Festinger and Carlsmith, 1959).

### From macro-valence at time *t* to micro-valence at time *t* + 1

The integration to macro-valence results in a loss of information because it reflects a “many-to-one mapping” (Cacioppo and Berntson, 1994, p. 412; Cacioppo et al., 1999); that is, combinations of micro-valences may predict a particular macro-valence, but a particular macro-valence corresponds to various micro-valence combinations. As a result, macro-valence is ambiguous with regard to the specific combination of underlying micro-valences. Furthermore, macro-valence may result from factors other than micro-valences, such as hormones. As such, there is no clear relation between macro-valence at time *t* to micro-valences at time *t* + 1.

However, individuals may misattribute their experienced general affect when making more specific evaluations (Schwarz and Clore, 1983). As a result, in their everyday life, individuals’ general macro-valence may influence more specific micro-valences. Indeed, in their research on affective experiences in daily life, Kuppens et al. (2012) found that core affect valence [operationalized as (un)pleasantness] influences subsequent appraisals of motive consistency, coping potential, other agency, and future expectancy.

### From micro-valence at time *t* to micro-valence at time *t* + 1

In addition to an influence of micro-valences on macro-valence, micro-valences also influence subsequent micro-valences (1) by facilitating the processing of particular appraisal criteria, (2) by providing constraints on the salience of other appraisal criteria, and (3) by increasing an appraisal result’s salience.

First, research on emotion-congruent processing suggests that information that is congruent with a previous appraisal criterion may be processed more efficiently. For example, sad individuals identified words faster and found arguments more persuasive that were sadness rather than anger congruent (Niedenthal et al., 1997). However, this research was conducted on the level of emotions and not appraisals. One hypothesis is that appraisal outcomes facilitate appraisal-congruent processing in subsequent situations. In the long run, a tendency to process situations with particular appraisal criteria may produce an appraisal bias (Scherer and Brosch, 2009).

Second, micro-valences resulting from one appraisal may constrain the salience of other appraisals. The CPM (Scherer, 2009) suggests that appraisal outcomes for some appraisal criteria are necessary before other appraisal criteria become relevant in a situation. Indeed, encephalographic and psychophysiological research suggests that pleasantness, goal conduciveness, and power are processed in a sequential order (Aue et al., 2007; Lanctôt and Hess, 2007; Grandjean and Scherer, 2008; Delplanque et al., 2009; Gentsch et al., submitted). For example, pleasantness is processed before goal conduciveness (Aue et al., 2007; Lanctôt and Hess, 2007; Grandjean and Scherer, 2008). More research is needed to understand how these and other appraisals constrain each other.

Finally, the appraisal tendency framework (Lerner and Keltner, 2000) suggests that appraisal results in one situation may be carried over to a new situation. For example, Lerner and Keltner (2001) showed that high control appraisal in one situation lead to the perception of high control in subsequent, unrelated situations. In their study, control and power appraisals were not clearly differentiated. A non-linear dynamic systems view suggests an underlying process for this carry-over effect in which evaluations at time *t* stabilize as a result of interactions among system elements; an activate evaluation can suppress competing evaluations (e.g., Zeeman, 1976).

Other research suggests that when an event ends abruptly, an opponent process may occur (Solomon and Corbit, 1974). With an opponent process, an initial evaluation (e.g., positive sensation) automatically triggers a contrasting evaluation (e.g., negative sensation) that lasts for several minutes or longer, followed by a return to homeostasis. When the pleasant sensation stops abruptly (e.g., interruption of sexual stimulation), the opponent process from the contrasting evaluation can result in a negative sensation. Similarly, when a situation appears to be goal obstructive (e.g., worry about an illness), the abrupt end of the situation (e.g., when a doctor says one does not have the illness) may not result in a neutral feeling, but in positive affect. Solomon and Corbit (1974) suggest that the opponent process may become stronger after repeated experiences, explaining the temporal dynamic of affective experiences ranging from drug addiction to parachute jumping. For example, the opponent process model has proven useful in research on drug addiction (e.g., Koob and Le Moal, 2008) and “addictive” pro-social behaviors (e.g., Piliavin et al., 1982).

Whether or not a micro-valence is carried over to a new situation or leads to an opponent process may depend on the ambiguity of the situation. One hypothesis is that if the new situation is ambiguous, a micro-valence may be carried over, but if it is not ambiguous, an opponent process may occur.

### From macro-valence at time *t* to macro-valence at time *t* + 1

As stated earlier, macro-valence is influenced by a variety of factors. This also includes previous macro-valence. There may be a homeostasis of macro-valence with individual differences in reported general negative versus positive affectivity. For example, neuroticism is characterized by high levels of negative affect and extraversion by high levels of positive affect (Costa and McCrae, 1980; Rusting and Larsen, 1997). Differences across individuals in baseline macro-valence, assessed with measures of well-being, are partially genetically determined (Lykken and Tellegen, 1996).

Also, context factors may increase the duration of macro-valences. For example, Wilson and colleagues found that uncertainty amplifies the duration and intensity of affective reactions to movies (Wilson et al., 2005; Bar-Anan et al., 2009). This effect does not appear to be driven by increased attention to the emotional event, but by increased engagement with it (Bar-Anan et al., 2009). Note that it is not clear whether the increased duration and intensity of mood is driven by specific micro-valences or occurs at the level of macro-valence.

### From micro-valence at time *t* to emotions at time *t* + 1

Valence is at the heart of emotional experiences (though valence is not limited to emotions, but also relevant for, e.g., attitudes and decision making), and consequentially, the relation of micro-valences and emotions should be discussed. Emotions are a subgroup of affect categories, which also include, for example, affective metaphors. Which category should be considered an “emotion” is a matter of debate (e.g., is love an emotion? Shaver et al., 1996; Scherer, 2005). Some categories can be expressed by using an emotion term, particularly those categories reflecting basic or modal emotions, such as anger, sadness, or joy (Ekman and Friesen, 1976; Scherer, 2009). Additionally or alternatively to emotion terms, affect categories may be communicated by natural or culturally learned spontaneous or enacted postures, gestures, and vocal and facial expressions. As research on emotions shows, more information about the individual and situation than the micro-valences discussed in this paper may be needed to specify an emotion. As reviewed earlier, emotions are typically considered multicomponential, including appraisals, action tendencies, subjective feelings, and physiological changes (e.g., Shuman and Scherer, in press).

On the one hand, there is considerable variability in emotion terms across ontology, individuals, cultures, and generations (e.g., Russell, 1991; Wierzbicka, 1997). Children express anger non-verbally before they are able to express it verbally. Also, individuals differ in how closely they monitor their affective state and in how much detail they express it (Gohm and Clore, 2000). For example, for some individuals self-reported negative emotions are strongly correlated, reflecting a more global concern with negative versus positive feelings, whereas for other individuals self-reported negative emotions correlate weakly, suggesting a more fine-grained monitoring of their affective states (Barrett, 2004). Furthermore, individuals may differ in how many appraisals they associate with a particular emotion (Kuppens and Tong, 2010). For example, anger is associated with a norm violation for only some individuals (Kuppens et al., 2007).

On the other hand, there are cultural similarities in the appraisal-emotion relation for many emotion terms (Scherer, 1997; Fontaine et al., in press) and systematic relations between appraisals and emotions across individuals (e.g., Frijda et al., 1989). Researchers continue to specify how individual appraisals relate to specific emotions (e.g., Smith and Ellsworth, 1985; Roseman et al., 1990; Smith et al., 1993; Scherer, 1997; Kuppens et al., 2003, 2007; Siemer et al., 2007; Parkinson and Roper, 2009; Tong et al., 2009).

From recent research on emotions, we conceptualize the relation between micro-valences and emotions as a non-linear dynamic system (Zeeman, 1976; Camras, 1992, 2011; Scherer, 2000; Thagard and Nerb, 2002; Lewis, 2005; Izard, 2007). Non-linear dynamic systems describe how elements of a system interact and self-organize by nested positive and negative feedback loops. So-called attractor states in the system reflect stable patterns that the system elements are drawn to. Affect categories characterized by affect emotion terms may correspond to such stable patterns. Micro-valences at time *t* may be drawn to the stable patterns reflecting a particular emotion at time *t* + 1, such as happiness.

Non-linear dynamic systems theory shares many features with Gestalt theory. Similar to gestalts, “what happens to a part of the whole is … determined by the laws of the inner structure of its whole,” and “what is happening in the whole cannot (always) be deduced from the characteristics of the separate pieces” (Wertheimer, 1944, p. 84). As the elements in a system constrain themselves over time, the status of one element can change the status of another element and vice versa. Analyses can focus on the emergence of the pattern from unstable configurations (path from micro-valences to emotions; appraisals can then be regarded as a process that drives the emergence of a particular emotion), or on the influence of the pattern on subsequent patterns (path from micro-valences at time *t* to micro-valences at time *t* + 1). As was observed by Wertheimer (1944), p. 87) for gestalts, “something may be altered in each component part and still the whole remains identical, or very little may be altered and the whole is completely changed.”

Non-linear dynamic systems theory may further specify the relation between parts and wholes, leading to new hypotheses. For example, Scherer, 2000; see also Sander et al., 2005) proposed, on the basis of a particular non-linear dynamic system, that with high-power appraisals, small changes in goal conduciveness appraisals may not change an emotion until a threshold is reached at which the emotion changes drastically. In contrast, with low-power appraisals, emotions may change more gradually. Furthermore, in addition to the appraisals elicited by a stimulus, the prior state of the individual’s appraisals may influence the appraisal-emotion relation (Scherer, 2000; Sander et al., 2005). With regard to micro-valences, this leads to the prediction that micro-valences at time *t* influence an emotion at time *t* + 1 depending on the state of the micro-valences at time *t* − 1.

The question of temporal dynamics is interesting with regard to the currently discussed micro-valences, but research on the question of temporal changes between emotions should probably go beyond these micro-valences. This is because emotions are multicomponential, including valenced and non-valenced appraisals, action tendencies, and other components. The five valenced appraisals discussed in this paper contribute to emotion categories, but they are not sufficient to fully describe emotions and their dynamics. Also, from a non-linear dynamic systems view, a change in any one emotion component (e.g., appraisal, physiological state, action tendency) could induce the change from one attractor state to another. For example, physiological changes (e.g., drug induced increase in heart rate) may drive changes in emotions (e.g., from neutral to fear) including the associated appraisals. In this example, appraisals would be regarded not as the process that drives the emotion, but as the mental contents associated with the emotion.

As discussed before, emotion terms are one of many ways to express an affective state. If systematic relations exist between underlying components (e.g., micro-valences) and emotion terms, then similar systematic relations may exist for other affect categories, such as affective metaphors. For example, one could examine whether, similar to sadness, “feeling blue” may be related to low power and moral goodness. Similar to emotion terms, metaphoric expressions may furthermore be systematically related to other emotion components (e.g., action tendencies).

### Macro-valence at time *t* moderates the paths from micro-valence at time *t* to emotions and macro-valence at time *t* + 1

Although macro-valence does not directly influence affect categories, one’s current level of macro-valence at time *t* may influence how micro-valences at time *t* are integrated to macro-valence or emotion at time *t* + 1. For example, research shows that individuals in a more negative affective state focus more on each individual evaluation and put more effort into integrating evaluations than do those in a less negative state (Luce et al., 1997). Thus, one would predict that with negative macro-valence at time *t*, the integration of micro-valences at time *t* to macro-valence at time *t* + 1 would follow a more effort-intense integration function. Similarly, a more effortful semantic categorization may lead to more fine-grained emotion expressions. In contrast, with positive macro-valence at time *t*, micro-valences at time *t* would be integrated to macro-valence at time *t* + 1 with a less effort-intense integration function. Similarly, a less effortful semantic categorization may lead to less fine-grained emotion expressions. Another open question is whether the influence of macro-valence is an effect of one’s general feeling of positivity or negativity that serves as information about processing requirements (Schwarz and Clore, 1983) or whether it could be better explained by particular associated appraisals (e.g., uncertainty; see From Micro-Valences at Time *t* to Macro-Valence at Time *t* + 1).

### No path from emotions at time *t* to macro-valence at time *t* + 1

It may seem surprising that we did not include a path from emotions to macro-valence, given the abundant research evidence showing that emotions are reliably structured along a valence dimension (Cacioppo and Berntson, 1994; Barrett and Russell, 1998; Watson et al., 1999). However, as reviewed earlier, theoretical analyses show that emotions cannot unambiguously be classified as positive or negative (Solomon and Stone, 2002; Charland, 2005; Colombetti, 2005; Zeelenberg and Pieters, 2006; Pfister and Böhm, 2008). Some researchers have proposed that only specific emotions can be unambiguously mapped to macro-valence (Pfister and Böhm, 2008). In contrast, we suggest that emotions may become the object of appraisals, and the appraisal outcomes may determine the extent to which emotions are experienced as positive or negative. For example, categorizing one’s own reaction as happiness at a funeral may lead to the micro-valence of moral badness and – depending on the importance given to moral badness relative to other appraisals in this situation – to negative macro-valence. In many situations, though, happiness is associated with positive micro-valences. As a result of previous experiences or cultural knowledge, there is a high overall likelihood that happiness is positive (the affective quality of happiness is positive; Russell, 2003). Consequently, emotions may be reliably identified as positive or negative, for example, in rating tasks, reflecting the knowledge about their affective quality rather than current macro-valence.

We propose to replace the old question, “are specific emotions positive or negative?” with the new question, “why is it that people say that an emotion is negative or positive?” Why is fear typically considered a negative emotion although it may be useful to avoid danger? Why is anger typically considered a negative emotion although “the arousal (e.g., feeling strong) may be experienced as pleasurable, and the consequences of expressing one’s anger (putting the other person in his place) may be quite enjoyable” (Pfister and Böhm, 2008, p. 7)? What is the underlying mechanism? What are the underlying micro-valences, and which integration rule links them to a particular macro-valence? Some suggest that a single micro-valence, e.g., the pleasantness of having the emotion, is not sufficient to understand why an emotion is seen as positive or negative (Deonna and Teroni, 2012), but more research is needed to understand the evaluation of emotions.

Identifying the link between appraisals and affective quality may also help to explain cross-cultural differences in evaluating emotions as more or less positive or negative. For example, Johnson et al. (1986) report that anger is a more negative emotion for the Machiguenga from southeastern Peru than for other cultures and explain the cultural differences by observing that anger is regarded as particularly disruptive of the social peace in this culture. This suggests that the moral badness of anger is more salient or weighted more heavily in this culture than in other cultures. However, direct studies of appraisals and emotions are needed to further examine the question of cultural differences in the evaluations of emotion and related micro-valences.

## Implications for Mixed Feelings and Choice

The framework suggested here incorporates the explanatory power of previous multifaceted and one-dimensional conceptualizations of valence. Similar to previous research on valence as the common currency, we construe one-dimensional macro-valence as a primary predictor for choice. As in appraisal theory (e.g., Scherer, 2001), we suggest that multifaceted valences can result in mixed feelings, and conflicts can arise either between levels of processing (e.g., a conflict between internalized and deliberately endorsed gender roles for the ought self) or between different micro-valences (e.g., a conflict between pleasantness and goal conduciveness appraisals).

Furthermore, a framework that conceptualizes valence at two levels suggests that conflict can result from consciously integrated micro-valences that do not correspond to one’s unconscious macro-valence. In other words, one’s reasoning about a situation does not correspond to one’s gut feeling about it. The source of a discrepant unconscious macro-valence may lie, firstly, in a different integration of micro-valences at the unconscious level. For example, a girl may appraise having a romantic partner as not particularly pleasurable, but congruent with her self-concept and morally desirable. Unconsciously, the low pleasantness micro-valence may be most important in determining her macro-valence, but consciously, the girl may be reluctant to justify her relationship status based on this presumably superficial criterion. She may then experience a conflict between the unconscious and the conscious macro-valence regarding this partner. This conflict may be evident, for example, in non-verbally avoiding and verbally approaching the partner. Research on implicit and explicit attitudes and motives is relevant to understand this type of conflict. This research suggests that individuals may simultaneously hold unconscious and conscious attitudes (e.g., Wilson et al., 2000). Secondly, conflicts between conscious and unconscious macro-valence may result from other influences on unconscious macro-valence that are more powerful than micro-valences. For example, hormones may influence macro-valence more strongly than micro-valences. Regulating persistent conflicts between conscious and unconscious macro-valence may consume volitional strength, which may lower well-being in the long run, as suggested by research on implicit and explicit motive discrepancies (Kehr, 2004).

Discrepancies of conscious and unconscious macro-valence may furthermore influence the quality of one’s choices. To stick with the example of the girl with an unpleasant partner, she may consciously tally the number of positive micro-valences but unconsciously apply a one-clever-cue heuristic. A conscious choice would be to remain with the partner, but an unconscious choice using her pleasantness appraisals as one-clever-cue would be to break up with him. Choices may reflect the conscious evaluation when cognitive resources are sufficiently high to retrieve it and when it is stronger than an unconscious evaluation of the same situation (Wilson et al., 2000). It depends on the particular situation which process yields a better outcome (e.g., Gigerenzer and Gaissmaier, 2011; Huizenga et al., 2012).

To summarize, by incorporating one-dimensional and multifaceted conceptions of valence, the current framework can take advantage of existing explanations for choice and mixed emotions. Furthermore, we propose additional pathways that lead to mixed emotions and to suboptimal choice.

## Conclusion

Despite the popularity of the valence concept, valence is differently defined in previous research as multifaceted (e.g., Scherer, 2010) or one-dimensional (e.g., Cabanac, 1992), and its application in affective science has repeatedly been criticized (Solomon and Stone, 2002; Colombetti, 2005). Rather than making a case for either multifaceted or one-dimensional valence alone, we suggested that they both have advantages and disadvantages and should be integrated. Here, we propose a new framework that distinguishes between two fundamental meanings of valence. With micro-valence, we refer to valenced appraisal outputs (pleasantness, goal conduciveness, power, self-congruence, moral goodness). In any situation, multiple appraisals may coexist, resulting in a multifaceted, potentially mixed positive-negative affective experience and influencing the nature of specific behavioral tendencies. Co-occurring with micro-valence, macro-valence is one-dimensional and predicts choice. Finally, for communication, affect categories may simultaneously exist, such as emotions. Multiple relations exist between micro- and macro-valence and affect categories across time.

Similarities to and differences from previous models of valence can be noted. First, similar to our framework, the evaluative space model (Cacioppo and Berntson, 1994; Cacioppo et al., 1997, 1999) and a three-level hierarchical model by Tellegen et al. (1999) emphasize the relation between underlying evaluations and macro-valence. These models focus on positive versus negative evaluations, whereas we distinguish qualitatively different micro-valences. Thus, our approach complements these models. Tellegen et al. (1999) describe the dimensional and hierarchical structure of affect, including affect categories, bivariate, and bipolar valence, derived from studies on affect ratings, whereas the evaluative space model discusses the processes underlying the integration of bivariate evaluations to bipolar evaluations. We add to this literature by discussing the temporal relation between valences at different levels and affect categories. In our framework, micro-valences, macro-valence, and emotion occur simultaneously. Furthermore, micro- and macro-valence influence each other across time. The dynamic between micro- and macro-valence can be described as a non-linear dynamic system. Non-linear dynamic systems are increasingly used to describe emotion experience, development, encoding, and decoding (e.g., Zeeman, 1976; Fogel and Thelen, 1987; Wolff, 1987; Camras, 1992, 2011; Thagard and Nerb, 2002; Lewis, 2005; Izard, 2007; Sacharin et al., 2012).

Second, our approach is also compatible with the Gestalt approach to emotions. For example, Castelfranchi and Miceli (2009) describe cognitive-motivational compounds of emotional experience as gestalts emerging from beliefs, evaluative beliefs, and desires. However, these authors focus on differentiating emotions in an approach reminiscent of Wierzbicka’s (1999) emotion scripts, in contrast to the focus of our framework on valence.

Third, our framework complements the framework for studying the neurobiology of value-based decision making by Rangel et al. (2008). The authors describe the steps in a decision-making process as the representation of a situation, valuation, action selection, outcome evaluation, and learning. The levels of valence discussed in our framework seem to be mostly relevant for the outcome evaluation. As Rangel et al. note (p. 8), “much remains to be understood about the outcome-valuation system. What network is responsible for computing positive and negative outcome values in different types of domains? How are positive and negative outcome-valuation signals integrated?” Distinguishing micro-valences on the basis of appraisals and examining their relation to macro-valence may be a starting point to examine this question.

The micro-valences discussed in this paper may additionally be systematically related to the valuation step in the decision-making process described by Rangel et al. (2008), e.g., to compute how pleasant or how goal conducive a situation is. To predict the value of a course of action, the authors distinguish three systems. With the Pavlovian system, value is assigned by evolution, with the habitual system by repeated stimulus-response associations and trial-and-error, and with the goal-directed system by computations of action-outcome associations. Possibly, specific value systems are more closely related to certain appraisals, such as pleasantness to the Pavlovian system, goal conduciveness to the goal-directed system, and self-congruency to the habitual system. Thus, Rangel et al.’s model complements the current framework because it can describe in great detail how the initial assessment of pleasantness, goal conduciveness, etc. comes about before different evaluations are integrated to guide choice, behavior, and communication as described in the current framework.

Finally, our suggestion that both a “common currency” and qualitatively different types of valence exist is highly consistent with recent neuroimaging research. The striatum, ventromedial prefrontal cortex, and orbitofrontal cortex have repeatedly been suggested as areas that code various types of valence (e.g., Shizgal and Conover, 1996; Schultz, 2000; Montague and Berns, 2002; Berridge, 2003; Saxe and Haushofer, 2008; Chib et al., 2009). At the same time, different types of valence have distinct neural correlates (e.g., Sescousse et al., 2010). For example, in addition to neural correlates of the subjective value across types of rewards in the ventromedial prefrontal cortex and the striatum, Levy and Glimcher (2011) identified distinct neural correlates of the subjective value of food rewards in the hypothalamus and of money rewards in the posterior cingulated cortex. While these authors focus on the qualitative differences in the reward value of different objects (food, money), however, we focus on the qualitative difference between perspectives with which the same object may be approached (e.g., pleasantness or goal conduciveness of food). In future research, one may want to manipulate the perspective with which objects, e.g., food, are approached, to more directly examine the neural correlates of the micro-valences discussed in the current paper.

Instead of debating the value of one-dimensional valence (Solomon and Stone, 2002; Colombetti, 2005) and whether one-dimensional valence or emotion is the more powerful concept to explain affective behavior (Barrett et al., 2007a; Izard, 2007; Panksepp, 2008), we suggest that researchers should focus on the relations between micro- and macro-valence and affect categories (e.g., emotions). For some of these relations, knowledge has already accumulated, in particular regarding the relation of micro-valences to emotions. For most paths, however, new and yet-to-be-tested hypotheses were derived.

The framework applies to more or less “emotional” evaluations; that is, the difference between cognition and emotion is purposefully underemphasized. Research on emotions, but also on emotion and cognition, as well as on behavioral economics, is relevant to the current framework, the latter particularly for the relation of micro-valences to macro-valence.

The confounding of affective and cognitive processes is an important difference between our model and Russell’s psychological constructionist model, which distinguishes between “cold” perceptual processes (affective quality) and “hot” core affect. In the psychological constructionist model, a lack of improvement of a depressed patient’s mood by viewing a pleasant sunset is used as a good example for the independence of an affective state (depression) from a cognitive perception (of the sunset; Russell, 2003). Also, the nature of mixed feelings is reduced to cognitive perceptions of affective quality. In contrast, in our framework, there is no problem to account for the occurrence of mixed feelings as well as mixed cognitions. This is more compatible with the current evidence on mixed feelings reviewed above. Furthermore, on the basis of our framework, we would explain the example of the depressed patient by referring to different appraisals rather than to “hot” and “cold” processes. Specifically, low goal conduciveness (depression) may not be ameliorated by a pleasant experience (sunset). This explanation fits better with the existing evidence that anhedonic individuals do not differ in their reported pleasure from consummatory behaviors (e.g., eating dinner), but report reduced anticipatory pleasure regarding goal-directed activities (e.g., making dinner; Gard et al., 2007). Indeed, affect regulation may be most powerful when targeting the appraisal that needs regulating, in the example, goal conduciveness. Further research is needed to examine these explanations.

A possible extension of the model that is to include a path from emotions at time *t* to emotions at time *t* + 1. For example, it has been suggested that language influences which affective states are recognized by shaping category learning and application (Barrett et al., 2007b). A particular category may become more salient based on the recent usage of this category, similar to a general priming effect (Carroll and Young, 2005). However, satiating the semantic meaning of a word by repeated exposure (e.g., 30 times) reduces categorization abilities for this term for affective stimuli (Lindquist et al., 2006). While this path adds to the completeness of the framework as depicted in Figure 2, it is not directly relevant to the discussion of valence.

Another possible extension of the framework is to include additional micro-valences. We examined only a limited number of appraisals derived from the CPM (Scherer, 1984, 2009). Future research may examine the affective experiences associated with other appraisals. With the appraisals examined in this paper, we implicitly assumed a linear increase in positivity (negativity) for each appraisal. For example, the more a situation is in line with the ideal self, the more moral goodness may be experienced. However, we can speculate that both low and high certainty may be aversive, but that medium levels of certainty may be positive. Also, the valence of certainty may be moderated by other appraisals. For example, low certainty about otherwise positive events may be experienced as positive, but low certainty about negative events may be particularly troubling (Bar-Anan et al., 2009).

We hope to have shown that although there is much about valence that we do not yet understand it can be investigated more accurately by adopting a new integrative framework. Given the problems with a one-dimensional view on valence, it has previously been suggested to “move beyond” (Zeelenberg and Pieters, 2006, p. 119) or to “abandon” (Solomon and Stone, 2002, p. 432) valence. On the contrary, we believe that valence has a promising future if investigated at its different levels.

## Conflict of Interest Statement

The authors declare that the research was conducted in the absence of any commercial or financial relationships that could be construed as a potential conflict of interest.

## References

[B1] Abelson R. P. Sermat V. (1962). Multidimensional scaling of facial expressions. J. Exp. Psychol. 63, 546–554 10.1037/h0042280 13858932

[B2] Acker F. (2008). New findings on unconscious versus conscious thought in decision making: additional empirical data and meta-analysis. Judgm. Decis. Mak. 3, 292–303

[B3] Anderson N. H. (1989). “Information integration approach to emotions and their measurement,” in Emotion: Theory, Research, and Experience, Vol. 4: The Measurement of Emotion, eds Plutchik R. Kellerman H. (New York: Academic Press), 133–186

[B4] Appadurai A. (1981). Gastro-politics in Hindu South Asia. Am. Ethnol. 8, 494–511 10.1525/ae.1981.8.3.02a00050

[B5] Aue T. Flykt A. Scherer K. R. (2007). First evidence for differential and sequential efferent effects of stimulus relevance and goal conduciveness appraisal. Biol. Psychol. 74, 347–357 10.1016/j.biopsycho.2006.09.001 17052833

[B6] Bandura A. (1977). Self-efficacy: toward a unifying theory of behavioral change. Psychol. Rev. 84, 191–215 10.1037/0033-295X.84.2.191 847061

[B7] Bänziger T. Tran V. Scherer K. R. (2005). “The emotion wheel. A tool for the verbal report of emotional reactions,” in Poster presented at the conference of the International Society of Research on Emotion, Bari

[B8] Bar-Anan Y. Wilson T. D. Gilbert D. T. (2009). The feeling of uncertainty intensifies affective reactions. Emotion 9, 123–127 10.1037/a0014607 19186925

[B9] Barrett L. F. (1996). Hedonic tone, perceived arousal, and item desirability: three components of self-reported mood. Cogn. Emot. 10, 47–68 10.1080/026999396380385

[B10] Barrett L. F. (2004). Feelings or words? Understanding the content in self-report ratings of experienced emotion. J. Pers. Soc. Psychol. 87, 266–281 10.1037/0022-3514.87.2.266 15301632 PMC1351136

[B11] Barrett L. F. (2006). Valence is a basic building block of emotional life. J. Res. Pers. 40, 35–55 10.1016/j.jrp.2005.08.006

[B12] Barrett L. F. Bliss-Moreau E. (2009). “Affect as a psychological primitive,” in Advances in Experimental Social Psychology, 1st Edn, Vol. 41, ed. Zanna M. P. (Burlington: Elsevier Inc.), 167–218 10.1016/S0065-2601(08)00404-8 PMC2884406 20552040

[B13] Barrett L. F. Lindquist K. A. Bliss-Moreau E. Duncan S. Mize J. Brennan L. (2007a). Of mice and men: natural kinds of emotions in the mammalian brain? A response to Panksepp and Izard. Perspect. Psychol. Sci. 2, 297–311 10.1111/j.1745-6916.2007.00046.x 19079552 PMC2597798

[B14] Barrett L. F. Lindquist K. A. Gendron M. (2007b). Language as context for the perception of emotion. Trends Cogn. Sci. (Regul. Ed.) 11, 327–332 10.1016/j.tics.2007.06.003 17625952 PMC2225544

[B15] Barrett L. F. Russell J. A. (1998). Independence and bipolarity in the structure of current affect. J. Pers. Soc. Psychol. 74, 967–984 10.1037/0022-3514.74.4.967

[B16] Baumeister R. F. Leary M. R. (1995). The need to belong: desire for interpersonal attachments as a fundamental human motivation. Psychol. Bull. 117, 497–529 10.1037/0033-2909.117.3.497 7777651

[B17] Berridge K. C. (2003). Pleasures of the brain. Brain Cogn. 52, 106–128 10.1016/S0278-2626(03)00014-9 12812810

[B18] Berridge K. C. Kringelbach M. L. (2008). Affective neuroscience of pleasure: reward in humans and animals. Psychopharmacology (Berl.) 199, 457–480 10.1007/s00213-008-1099-6 18311558 PMC3004012

[B19] Berridge K. C. Valenstein E. S. (1991). What psychological process mediates feeding evoked by electrical stimulation of the lateral hypothalamus? Behav. Neurosci. 105, 3–14 10.1037/0735-7044.105.1.3 2025391

[B20] Bless H. Schwarz N. Clore G. L. Golisano V. Rabe C. Wölk M. (1996). Mood and the use of scripts: does a happy mood really lead to mindlessness? J. Pers. Soc. Psychol. 71, 665–679 10.1037/0022-3514.71.4.665 8888596

[B21] Block J. (1957). Studies in the phenomenology of emotions. J. Abnorm. Psychol. 54, 358–363 10.1037/h0040768 13448842

[B22] Bodenhausen G. V. Sheppard L. A. Kramer G. P. (1994). Negative affect and social judgment: the differential impact of anger and sadness. Eur. J. Soc. Psychol. 24, 45–62 10.1002/ejsp.2420240104

[B23] Booth D. A. Higgs S. Schneider J. Klinkenberg I. (2010). Learned liking versus inborn delight: can sweetness give sensual pleasure or is it just motivating? Psychol. Sci. 20, 1–8 10.1177/0956797610385356 20921573

[B24] Bradley M. Lang P. J. (1994). Measuring emotion: the self-assessment manikin and the semantic differential. J. Behav. Ther. Exp. Psychiatry 25, 49–59 10.1016/0005-7916(94)90063-9 7962581

[B25] Bridges K. M. B. (1932). Emotional development in early infancy. Child Dev. 3, 324–341 10.2307/1125359

[B26] Bush L. E. (1973). Individual differences multidimensional scaling of adjectives denoting feelings. J. Pers. Soc. Psychol. 25, 50–57 10.1037/h0034274 4688168

[B27] Cabanac M. (1979). Sensory pleasure. Q. Rev. Biol. 54, 1–29 10.1086/410981 379894

[B28] Cabanac M. (1985). Preferring for pleasure. Behav. Biol. 42, 1151–1155 10.1093/ajcn/42.5.1151 4061361

[B29] Cabanac M. (1986). Money versus pain: experimental study of a conflict in humans. J. Exp. Anal. Behav. 46, 37–44 10.1901/jeab.1986.46-37 3746186 PMC1348254

[B30] Cabanac M. (1992). Pleasure: the common currency. J. Theor. Biol. 155, 173–200 10.1016/S0022-5193(05)80594-6 12240693

[B31] Cabanac M. (1995). Palatability vs. money: experimental study of a conflict of motivations. Appetite 25, 43–49 10.1006/appe.1995.0040 7495326

[B32] Cacioppo J. T. Berntson G. G. (1994). Relationship between attitudes and evaluative space: a critical review with emphasis on the separability of positive and negative substrates. Psychol. Bull. 115, 401–423 10.1037/0033-2909.115.3.401

[B33] Cacioppo J. T. Gardner W. L. Berntson G. G. (1997). Beyond bipolar conceptualizations and measures: the case of attitudes and evaluative space. Pers. Soc. Psychol. Rev. 1, 3–25 10.1207/s15327957pspr0101_2 15647126

[B34] Cacioppo J. T. Gardner W. L. Berntson G. G. (1999). The affect system has parallel and integrative processing components: form follows function. J. Pers. Soc. Psychol. 76, 839–855 10.1037/0022-3514.76.5.839

[B35] Camras L. A. (1992). Expressive development and basic emotions. Cogn. Emot. 6, 269–283 10.1080/02699939208411072

[B36] Camras L. A. (2011). Differentiation, dynamical integration and functional emotional development. Emot. Rev. 3, 138–146 10.1177/1754073910387944

[B37] Carroll N. C. Young A. W. (2005). Priming of emotion recognition. Q. J. Exp. Psychol. A. 58, 1173–1197 16194954 10.1080/02724980443000539

[B38] Carver C. S. Harmon-Jones E. (2009). Anger in an approach-related affect: evidence and implications. Psychol. Bull. 135, 183–204 10.1037/a0015026 19254075

[B39] Castelfranchi C. Miceli M. (2009). The cognitive-motivational compound of emotional experience. Emot. Rev. 1, 223–231 10.1177/1754073909103590

[B40] Charland L. C. (2005). The heat of emotion. Valence and the demarcation problem. J. Conscious. Stud. 12, 82–102

[B41] Chib V. S. Rangel A. Shimojo S. O’Doherty J. P. (2009). Evidence for a common representation of decision values for dissimilar goods in human ventromedial prefrontal cortex. J. Neurosci. 29, 12315–12320 10.1523/JNEUROSCI.5856-08.2009 19793990 PMC6666137

[B42] Colombetti G. (2005). Appraising valence. J. Conscious. Stud. 12, 103–126

[B43] Costa P. T. McCrae R. R. (1980). Influence of extraversion and neuroticism on subjective well-being: happy and unhappy people. J. Pers. Soc. Psychol. 38, 668–678 10.1037/0022-3514.38.4.668 7381680

[B44] Cramer L. Antonides G. (2011). Endowment effects for hedonic and utilitarian food products. Food Qual. Prefer. 22, 3–10 10.1016/j.foodqual.2010.05.020

[B45] Davidson R. J. (1994). Asymmetric brain function, affective style, and psychopathology: the role of early experience and plasticity. Dev. Psychopathol. 6, 741–758 10.1017/S0954579400004764

[B46] Delplanque S. Grandjean D. Chrea C. Coppin G. Aymard L. Cayeux I. (2009). Sequential unfolding of novelty and pleasantness appraisals of odors: evidence from facial electromyography and autonomic reactions. Emotion 9, 316–328 10.1037/a0015369 19485609

[B47] Deonna J. A. Teroni F. (2012). The Emotions: A Philosophical Introduction. Abingdon: Routledge

[B48] Diener E. Iran-Nejad A. (1986). The relationship in experience between various types of affect. J. Pers. Soc. Psychol. 50, 1031–1038 10.1037/0022-3514.50.5.1031

[B49] Drewnowski A. (1997). Taste preferences and food intake. Ann. Rev. Nutr. 17, 237–253 10.1146/annurev.nutr.17.1.237 9240927

[B50] Ekman P. Friesen W. V. (1976). Pictures of Facial Affect. Palo Alto, CA: Consulting Psychologists Press

[B51] Eliashberg J. Sawhney M. S. (1994). Modeling goes to Hollywood: predicting individual differences in movie enjoyment. Manage. Sci. 40, 1151–1173 10.1287/mnsc.40.9.1151

[B52] Ellsworth P. C. Scherer K. R. (2003). “Appraisal processes in emotion,” in Handbook of Affective Sciences, eds Davidson R. J. Goldsmith H. H. Scherer K. R. (New York: Oxford University Press), 572–595

[B53] Evans J. S. (2008). Dual-processing accounts of reasoning, judgment, and social cognition. Annu. Rev. Psychol. 59, 255–278 10.1146/annurev.psych.59.103006.093629 18154502

[B54] Feldman L. A. (1995). Variations in the circumplex structure of mood. Pers. Soc. Psychol. Bull. 21, 806–817 10.1177/0146167295218003

[B55] Festinger L. Carlsmith J. M. (1959). Cognitive consequences of forced compliance. J. Abnorm. Psychol. 58, 203–210 10.1037/h0041593 13640824

[B56] Fogel A. Thelen E. (1987). Development of early expressive and communicative action: reinterpreting the evidence from a dynamic systems perspective. Dev. Psychol. 23, 747–761 10.1037/0012-1649.23.6.747

[B57] Fontaine J. R. J. Scherer K. R. Roesch E. B. Ellsworth P. C. (2007). The world of emotions is not two-dimensional. Psychol. Sci. 18, 1050–1057 10.1111/j.1467-9280.2007.02024.x 18031411

[B58] Fontaine J. R. J. Scherer K. R. Soriano C. (in press). Components of Emotional Meaning: A Sourcebook. Oxford: Oxford University Press

[B59] Forgas J. P. (1992). On mood and peculiar people: affect and person typicality in impression formation. J. Pers. Soc. Psychol. 62, 863–875 10.1037/0022-3514.62.5.863

[B60] Forgas J. P. Fiedler K. (1996). Us and them: mood effects on intergroup discrimination. J. Pers. Soc. Psychol. 70, 28–40 10.1037/0022-3514.70.1.28

[B61] Freud S. (1920). A General Introduction to Psychoanalysis. Garden City, NY: Garden City Publishing Company Inc

[B62] Frijda N. Scherer K. R. (2009). “Affect (psychological perspective),” in The Oxford Companion to Emotion and the Affective Sciences, eds Sander D. Scherer K. R. (Oxford: Oxford University Press), 10

[B63] Frijda N. H. (2009). Emotions, individual differences and time course: reflections. Cogn. Emot. 23, 1444–1461 10.1080/02699930903093276

[B64] Frijda N. H. Kuipers P. ter Schure E. (1989). Relations among emotion, appraisal, and emotional action readiness. J. Pers. Soc. Psychol. 57, 212–228 10.1037/0022-3514.57.2.212

[B65] Gard D. E. Kring A. M. Gard M. G. Horan W. P. Green M. F. (2007). Anhedonia in schizophrenia: distinctions between anticipatory and consummatory pleasure. Schizophr. Res. 93, 253–260 10.1016/j.schres.2007.03.008 17490858 PMC1986826

[B67] Gigerenzer G. Gaissmaier W. (2011). Heuristic decision making. Annu. Rev. Psychol. 62, 451–482 10.1146/annurev-psych-120709-145346 21126183

[B68] Giner-Sorolla R. (1999). “Affect in attitude. Immediate and deliberative perspectives,” in Dual Process Theories in Social Psychology, eds Chaiken S. Trope Y. (New York: Guilford), 441–461

[B69] Gohm C. L. Clore G. L. (2000). Individual differences in emotional experience: mapping available scales to processes. Pers. Soc. Psychol. Bull. 26, 679–697 10.1177/0146167200268004

[B70] Goldman B. M. Kernis M. H. (2002). Role of authenticity in healthy psychological functioning and subjective well-being. Ann. Am. Psychother. Assoc. 5, 18–20

[B71] Grandjean D. Scherer K. R. (2008). Unpacking the cognitive architecture of emotion processes. Emotion 8, 341–351 10.1037/1528-3542.8.3.341 18540750

[B72] Green R. S. Cliff N. (1975). Multidimensional comparisons of structures of vocally and facially expressed emotion. Percept. Psychophys. 17, 429–438 10.3758/BF03203289

[B73] Hammond J. S. Keeney R. L. Raiffa H. (1998). Even swaps: a rational method for making trade-offs. Harv. Bus. Rev. 76, 137–149 10177863

[B74] Hanselmann M. Tanner C. (2008). Taboos and conflicts in decision making: sacred values, decision difficulty, and emotions. Judgm. Decis. Mak. 3, 51–63

[B75] Higgins E. T. (1987). Self-discrepancy: a theory relating self and affect. Psychol. Rev. 94, 319–340 10.1037/0033-295X.94.3.319 3615707

[B76] Huizenga H. M. Wetzels R. van Ravenzwaaij D. Wagenmakers E.-J. (2012). Four empirical tests of Unconscious Thought Theory. Organ. Behav. Hum. Decis. Process 117, 332–340 10.1016/j.obhdp.2011.11.010

[B77] Ito T. Cacioppo J. (2005). Variations on a human universal: individual differences in positivity offset and negativity bias. Cogn. Emot. 19, 1–26 10.1080/02699930441000120

[B78] Izard C. E. (2007). Basic emotions, natural kinds, emotion schemas, and a new paradigm. Perspect. Psychol. Sci. 2, 260–280 10.1111/j.1745-6916.2007.00053.x 26151969

[B79] Johnson A. Johnson O. Baksh M. (1986). The colors of emotions in Machiguenga. Am. Anthropol. 88, 674–681 10.1525/aa.1986.88.4.02a00700

[B80] Kahneman D. Tversky A. (1979). Prospect theory: an analysis of decision under risk. Econometrica 47, 263–292 10.2307/1914185

[B81] Kahneman D. Wakker P. P. Sarin R. (1997). Back to Bentham? Explorations of experienced utility. Q. J. Econ. 112, 375–405 10.1162/003355397555235

[B82] Keeney R. L. Raiffa H. (1976). Decisions with Multiple Objectives: Preferences and Value Trade-Offs. New York: John Wiley & Sons

[B83] Kehr H. M. (2004). Implicit/explicit motive discrepancies and volitional depletion among managers. Pers. Soc. Psychol. Bull. 30, 315–327 10.1177/0146167203256967 15030623

[B84] Keltner D. Gruenfeld D. H. Anderson C. (2003). Power, approach, and inhibition. Psychol. Rev. 110, 265–284 10.1037/0033-295X.110.2.265 12747524

[B85] Keskitalo K. Knaapila A. Kallela M. Palotie A. Wessman M. Sammalisto S. (2007). Sweet taste preferences are partly genetically determined: identification of a trait locus on chromosome 16.1-3. Am. J. Clin. Nutr. 86, 55–63 17616763 10.1093/ajcn/86.1.55

[B86] Koob G. F. Le Moal M. (2008). Addiction and the brain antireward system. Annu. Rev. Psychol. 59, 29–53 10.1146/annurev.psych.59.103006.093548 18154498

[B87] Kring A. M. Barrett L. F. Gard D. E. (2003). On the broad applicability of the affective circumplex: representations of affective knowledge among schizophrenia patients. Psychol. Sci. 14, 207–214 10.1111/1467-9280.02433 12741742

[B89] Kuppens P. Champagne D. Tuerlinckx F. (2012). The dynamic interplay between appraisal and core affect in daily life. Front. Psychol. 3:380 10.3389/fpsyg.2012.00380 23060842 PMC3466066

[B90] Kuppens P. Tong E. M. W. (2010). An appraisal account of individual differences in emotional experience. Soc. Personal. Psychol. Compass 4, 1138–1150 10.1111/j.1751-9004.2010.00324.x

[B88] Kuppens P. Van Mechelen I. Smits D. J. M. De Boeck P. (2003). The appraisal basis of anger: specificity, necessity and sufficiency of components. Emotion 3, 254–269 10.1037/1528-3542.3.3.254 14498795

[B91] Kuppens P. Van Mechelen I. Smits D. J. M. De Boeck P. Ceulemans E. (2007). Individual differences in patterns of appraisal and anger experience. Cogn. Emot. 21, 689–713 10.1080/02699930600859219

[B92] Lanctôt N. Hess U. (2007). The timing of appraisals. Emotion 7, 207–212 10.1037/1528-3542.7.1.207 17352576

[B93] Larsen J. T. McGraw A. P. (2011). Further evidence for mixed emotions. J. Pers. Soc. Psychol. 100, 1095–1110 10.1037/a0021846 21219075

[B94] Larsen J. T. McGraw A. P. Cacioppo J. T. (2001). Can people feel happy and sad at the same time? J. Pers. Soc. Psychol. 81, 684–696 10.1037/0022-3514.81.4.684 11642354

[B95] Lerner J. S. Keltner D. (2000). Beyond valence: toward a model of emotion-specific influences on judgment and choice. Cogn. Emot. 14, 473–493 10.1080/026999300402763

[B96] Lerner J. S. Keltner D. (2001). Fear, anger, and risk. J. Pers. Soc. Psychol. 81, 146–159 10.1037/0022-3514.81.1.146 11474720

[B97] Leventhal H. Scherer K. R. (1987). The relationship of emotion to cognition: a functional approach to a semantic controversy. Cogn. Emot. 1, 3–28 10.1080/02699938708408361

[B98] Levy D. J. Glimcher P. W. (2011). Comparing apples and oranges: using reward-specific and reward-general subjective value representation in the brain. J. Neurosci. 31, 14693–14707 10.1523/JNEUROSCI.3214-10.2011 21994386 PMC3763520

[B99] Lewin K. (1951). Field Theory in Social Science. Selected Theoretical Papers, (Westport, CT: Greenwood Press).

[B100] Lewis M. D. (2005). Bridging emotion theory and neurobiology through dynamic systems modeling. Behav. Brain Sci. 28, 169–194 10.1017/S0140525X0500004X 16201458

[B101] Lindeman M. Stark K. (1999). Pleasure, pursuit of health or negotiation of identity? Personality correlates of food choice motives among young and middle-aged women. Appetite 33, 141–161 10.1006/appe.1999.0241 10447986

[B102] Lindquist K. A. Barrett L. F. Bliss-Moreau E. Russell J. A. (2006). Language and the perception of emotion. Emotion 6, 125–138 10.1037/1528-3542.6.1.125 16637756

[B103] Litt A. Khan U. Shiv B. (2010). Lusting while loathing: parallel counterdriving of wanting and liking. Psychol. Sci. 21, 118–125 10.1177/0956797609355633 20424032

[B104] Luce M. F. Bettman J. R. Payne J. W. (1997). Choice processing in emotionally difficult decisions. J. Exp. Psychol. 23, 384–405 10.1037//0278-7393.23.2.384 9080010

[B105] Lykken D. Tellegen A. (1996). Happiness is a stochastic phenomenon. Psychol. Sci. 7, 186–189 10.1111/j.1467-9280.1996.tb00355.x

[B106] Markus H. R. Kitayama S. (1991). Culture and the self: implications for cognition, emotion, and motivation. Psychol. Rev. 98, 224–253 10.1037/0033-295X.98.2.224

[B107] McFarland D. J. Sibly R. M. (1975). The behavioural final common path. Philos. Trans. R. Soc. Lond. B Biol. Sci. 270, 265–293 10.1098/rstb.1975.0009 239416

[B108] McNamara J. M. Houston A. I. (1986). The common currency for behavioral decisions. Am. Nat. 127, 358–378 10.1086/284489

[B109] Mellers B. (2000). Choice and the relative pleasure of consequences. Psychol. Bull. 126, 910–924 10.1037/0033-2909.126.6.910 11107882

[B110] Miller G. E. (2004). Frontier masculinity in the oil industry: the experience of women engineers. Gend. Work Organ. 11, 47–73 10.1111/j.1468-0432.2004.00220.x

[B111] Miller N. E. (1959). “Liberalization of basic S-R concepts: extensions to conflict behavior, motivation, and social learning,” in Psychology: A study of a Science, ed. Koch S. (New York: McGraw-Hill), 198–292

[B112] Montague P. R. Berns G. S. (2002). Neural economics and the biological substrates of valuation. Neuron 36, 265–284 10.1016/S0896-6273(02)00974-1 12383781

[B113] Moors A. De Houwer J. (2006). Automaticity: a theoretical and conceptual analysis. Psychol. Bull. 132, 297–326 10.1037/0033-2909.132.2.297 16536645

[B114] Niedenthal P. M. Setterlund M. B. Halberstadt J. B. (1997). Being happy and seeing happy: emotional state mediates visual word recognition. Cogn. Emot. 11, 403–432 10.1080/026999397379863

[B115] Oceja L. Carrera P. (2009). Beyond a single pattern of mixed emotional experience. Eur. J. Psychol. Assess. 25, 58–67 10.1027/1015-5759.25.1.58

[B116] Ortony A. Clore G. L. Collins A. (1990). The Cognitive Structure of Emotions. Cambridge: Cambridge University Press

[B117] Panksepp J. (2008). Cognitive conceptualism – where have all the affects gone? Perspect. Psychol. Sci. 3, 305–308 10.1111/j.1745-6924.2008.00081.x 26158950

[B118] Parkinson B. Roper A. (2009). Appraisal ratings in diary reports of reasonable and unreasonable anger. Eur. J. Soc. Psychol. 39, 82–87 10.1002/ejsp.470

[B119] Payne J. W. Bettman J. R. Luce M. F. (1996). When time is money: decision behavior under opportunity-cost time pressure. Organ. Behav. Hum. Decis. Process 66, 131–152 10.1006/obhd.1996.0044

[B120] Peters E. Västfjäll D. Gärling T. Slovic P. (2006). Affect and decision making: a “hot” topic. J. Behav. Decis. Mak. 19, 79–85 10.1002/bdm.528

[B121] Pfister H.-R. Böhm G. (2008). The multiplicity of emotions: a framework of emotional functions in decision making. Judgm. Decis. Mak. 3, 5–17

[B122] Phelan S. (2002). Fads and fashions: the price women pay. Prim. Care Update Ob Gyns 9, 138–143 10.1016/S1068-607X(02)00105-1

[B123] Piliavin J. A. Callero P. L. Evans D. E. (1982). Addiction to altruism? Opponent-process theory and habitual blood donation. J. Pers. Soc. Psychol. 43, 1200–1213 10.1037/0022-3514.43.6.1200

[B124] Ramirez I. (1990). Why do sugars taste good? Neurosci. Biobehav. Rev. 14, 125–134 10.1016/S0149-7634(05)80213-1 2348939

[B125] Rangel A. Camerer C. Montague P. R. (2008). A framework for studying the neurobiology of value-based decision making. Nat. Rev. Neurosci. 9, 545–556 10.1038/nrn2357 18545266 PMC4332708

[B126] Reisenzein R. (1994). Pleasure-arousal theory and the intensity of emotions. J. Pers. Soc. Psychol. 67, 525–539 10.1037/0022-3514.67.3.525

[B127] Rimé B. (2009). Emotion elicits the social sharing of emotion: theory and empirical review. Emot. Rev. 1, 60–85 10.1177/1754073908097189

[B128] Roseman I. J. Spindel M. S. Jose P. E. (1990). Appraisals of emotion-eliciting events: testing a theory of discrete emotions. J. Pers. Soc. Psychol. 59, 899–915 10.1037/0022-3514.59.5.899

[B129] Russell J. A. (1978). Evidence of convergent validity on the dimensions of affect. J. Pers. Soc. Psychol. 36, 1152–1168 10.1037/0022-3514.36.10.1152

[B130] Russell J. A. (1980). A circumplex model of affect. J. Pers. Soc. Psychol. 39, 1161–1178 10.1037/h0077714

[B131] Russell J. A. (1991). Culture and the categorization of emotions. Psychol. Bull. 110, 426–450 10.1037/0033-2909.110.3.426 1758918

[B132] Russell J. A. (2003). Core affect and the psychological construction of emotion. Psychol. Rev. 110, 145–172 10.1037/0033-295X.110.1.145 12529060

[B133] Russell J. A. Bullock M. (1985). Multidimensional scaling of emotional facial expressions: similarity from preschoolers to adults. J. Pers. Soc. Psychol. 48, 1290–1298 10.1037/0022-3514.48.5.1290

[B134] Russell J. A. Lewicka M. Niit T. (1989). A cross-cultural study of a circumplex model of affect. J. Pers. Soc. Psychol. 57, 848–856 10.1037/0022-3514.57.5.848

[B135] Russell J. A. Mehrabian A. (1977). Evidence for a three-factor theory of emotions. J. Res. Pers. 11, 273–294 10.1016/0092-6566(77)90037-X

[B136] Russell J. A. Ridgeway D. (1983). Dimensions underlying children’s emotion concepts. Dev. Psychol. 19, 795–804 10.1037/0012-1649.19.6.795

[B137] Russell J. A. Steiger J. H. (1982). The structure in persons’ implicit taxonomy of emotions. J. Res. Pers. 16, 447–469 10.1016/0092-6566(82)90005-8

[B138] Rusting C. L. Larsen R. J. (1997). Extraversion, neuroticism, and susceptibility to positive and negative affect: a test of two theoretical models. Pers. Individ. Dif. 22, 607–612 10.1016/S0191-8869(96)00246-2

[B139] Sacharin V. Lee F. Gonzalez R. (2009). Identities in harmony: gender-work identity integration moderates frame switching in cognitive processing. Psychol. Women Q. 33, 275–284 10.1111/j.1471-6402.2009.01500.x

[B140] Sacharin V. Sander D. Scherer K. R. (2012). The perception of changing emotion expressions. Cogn. Emot. 26, 1273–1300 10.1080/02699931.2012.656583 22550942

[B141] Sander D. Grandjean D. Scherer K. R. (2005). A systems approach to appraisal mechanisms in emotion. Neural. Netw. 18, 317–352 10.1016/j.neunet.2005.03.001 15936172

[B142] Saxe R. Haushofer J. (2008). For love or money: a common neural currency for social and monetary reward. Neuron 58, 164–165 10.1016/j.neuron.2008.04.005 18439400

[B143] Scherer K. R. (1997). Profiles of emotion-antecedent appraisal: testing theoretical predictions across cultures. Cogn. Emot. 11, 113–150 10.1080/026999397379962

[B144] Scherer K. R. (1984). “On the nature and function of emotion: a component process approach,” in Approaches to Emotion, eds Scherer K. R. Ekman P. (Hillsdale, NJ: Lawrence Erlbaum Associates, Inc), 293–317

[B145] Scherer K. R. (2000). “Emotions as episodes of subsystem synchronization driven by nonlinear appraisal processes,” in Emotion, Development, and Self-Organization. Dynamic Systems Approaches to Emotion Development, eds Lewis M. D. Granic I. (Cambridge: Cambridge University Press), 70–99

[B146] Scherer K. R. (2001). “Appraisal considered as a process of multilevel sequential checking,” in Appraisal Processes in Emotion, eds Scherer K. R. Schorr A. Johnstone T. (New York: Oxford University Press), 92–120

[B147] Scherer K. R. (2004). “Feelings integrate the central representation of appraisal-driven response organization in emotion,” in Feelings and Emotions: The Amsterdam Symposium, eds Manstead A. S. R. Frijda N. H. Fischer A. H. (Cambridge: Cambridge University Press), 136–157

[B148] Scherer K. R. (2005). What are emotion? And how can they be measured? Soc. Sci. Inform. 44, 695–729 10.1177/0539018405058216

[B149] Scherer K. R. (2009). The dynamic architecture of emotion: evidence for the component process model. Emotion 23, 1307–1351 10.1080/02699930902928969

[B150] Scherer K. R. (2010). “The component process model: a blueprint for a comprehensive computational model of emotion,” in Blueprint for Affective Computing: A Sourcebook, eds Scherer K. R. Bänziger T. Roesch E. B. (Oxford: Oxford University Press), 47–70

[B151] Scherer K. R. Brosch T. (2009). Culture-specific appraisal biases contribute to emotion dispositions. Eur. J. Pers. 23, 265–288 10.1002/per.714

[B152] Scherer K. R. Ceschi G. (1997). Lost luggage: a field study of emotion-antecedent appraisal. Motiv. Emot. 21, 211–235 10.1023/A:1024498629430

[B153] Scherer K. R. Wranik T. Sangsue J. Tran V. Scherer U. (2004). Emotions in everyday life: probability of occurrence, risk factors, appraisals and reaction patterns. Soc. Sci. Inform. 43, 499–570 10.1177/0539018404047701

[B154] Schimmack U. (2001). Pleasure, displeasure, and mixed feelings: are semantic opposites mutually exclusive? Psychology 15, 81–97

[B155] Schimmack U. (2005). Response latencies of pleasure and displeasure ratings: further evidence for mixed feelings. Cogn. Emot. 19, 671–691 10.1080/02699930541000020

[B156] Schlosberg H. (1954). Three dimensions of emotion. Psychol. Rev. 61, 81–88 10.1037/h0054570 13155714

[B157] Schultz W. (2000). Multiple reward signals in the brain. Nat. Rev. Neurosci. 1, 199–207 10.1038/35043127 11257908

[B158] Schwartz S. H. Bardi A. (2001). Value hierarchies across cultures: taking a similarities perspective. J. Cross Cult. Psychol. 32, 268–290 10.1177/0022022101032003002

[B159] Schwarz N. Clore G. L. (1983). Mood, misattribution, and judgments of well-being: informative and directive functions of affective states. J. Pers. Soc. Psychol. 45, 513–523 10.1037/0022-3514.45.3.513

[B160] Seo M.-G. Bartunek J. M. Barrett L. F. (2010). The role of affective experience in work motivation: test of a conceptual model. J. Organ. Behav. 31, 951–968 21785527 10.1002/job.655 PMC3141585

[B161] Sescousse G. Redoute J. Dreher J.-C. (2010). The architecture of reward value coding in the human orbitofrontal cortex. J. Neurosci. 30, 13095–13104 10.1523/JNEUROSCI.3501-10.2010 20881127 PMC6633499

[B162] Shaver P. R. Morgan H. J. Wu S. (1996). Is love a “basic” emotion? Pers. Relatsh. 3, 81–96 10.1111/j.1475-6811.1996.tb00105.x

[B163] Shizgal P. Conover K. (1996). On the neural computation of utility. Psychol. Sci. 5, 37–43

[B164] Shuman V. Scherer K. R. (in press). “Concept and the structure of emotion,” in Handbook of Emotions in Education, eds Perkun R. Linnenbrink-Garcia L. (London: Routledge).

[B165] Siemer M. Mauss I. Gross J. J. (2007). Same situation – different emotions: how appraisals shape our emotions. Emotion 7, 592–600 10.1037/1528-3542.7.1.26 17683215

[B166] Smith C. A. Ellsworth P. C. (1985). Patterns of cognitive appraisal in emotion. J. Pers. 48, 813–838 10.1037/0022-3514.48.4.813 3886875

[B167] Smith C. A. Haynes K. N. Lazarus R. S. Pope L. K. (1993). In search of the “hot” cognitions: attributions, appraisals, and their relation to emotion. J. Pers. Soc. Psychol. 65, 916–929 10.1037/0022-3514.65.5.916 8246115

[B168] Solomon R. C. Stone L. D. (2002). On “positive” and “negative” emotions. J. Theory. Soc. Behav. 32, 417–435 10.1111/1468-5914.00180

[B169] Solomon R. L. Corbit J. D. (1974). An opponent-process theory of motivation: I. Temporal dynamics of affect. Psychol. Rev. 81, 119–145 10.1037/h0036128 4817611

[B170] Steele C. M. Liu T. J. (1983). Dissonance processes as self-affirmation. J. Pers. Soc. Psychol. 45, 5–19 10.1037/0022-3514.45.1.5

[B171] Steiner J. E. Glaser D. Hawilo M. E. Berridge K. C. (2001). Comparative expression of hedonic impact: affective reactions to taste by human infants and other primates. Neurosci. Biobehav. Rev. 25, 53–74 10.1016/S0149-7634(00)00051-8 11166078

[B172] Swann W. T. Read S. J. (1981). Self-verification processes: how we sustain our self-conceptions. J. Exp. Soc. Psychol. 17, 351–372 10.1016/0022-1031(81)90043-3

[B173] Tamir M. (2009). What do people want to feel and why? Psychol. Sci. 18, 101–105

[B174] Tellegen A. Watson D. Clark L. A. (1999). On the dimensional and hierarchical structure of affect. Psychol. Sci. 10, 297–303 10.1111/1467-9280.00159

[B175] Thagard P. Nerb J. (2002). Emotional gestalts: appraisal, change, and the dynamics of affect. Pers. Soc. Psychol. Rev. 6, 274–282 10.1207/S15327957PSPR0604_02

[B176] Tiedens L. Z. (2001). The effect of anger on the hostile inferences of aggressive and nonaggressive people: specific emotions, cognitive processing, and chronic accessibility. Motiv. Emot. 25, 233–251 10.1023/A:1012224507488

[B177] Tiedens L. Z. Linton S. (2001). Judgment under emotional certainty and uncertainty: the effects of specific emotions on information processing. J. Pers. Soc. Psychol. 81, 973–988 10.1037/0022-3514.81.6.973 11761319

[B178] Tong E. M. W. Ellsworth P. C. Bishop G. D. (2009). An s-shaped relationship between changes in appraisals and changes in emotions. Emotion 9, 821–837 10.1037/a0017812 20001125

[B179] Tversky A. (1972). Elimination by aspects: a theory of choice. Psychol. Rev. 79, 281–299 10.1037/h0032955

[B180] Valdez P. Mehrabian A. (1994). Effects of color on emotions. J. Exp. Psychol. 123, 394–409 10.1037/0096-3445.123.4.394 7996122

[B181] van Harreveld F. van der Pligt J. de Liver Y. N. (2009). The agony of ambivalence and ways to resolve it: introducing the MAID model. Pers. Soc. Psychol. Rev. 13, 45–61 10.1177/1088868308324518 19144904

[B182] Van Reekum C. Johnstone T. Banse R. Etter A. Wehrle T. Scherer K. (2004). Psychophysiological responses to appraisal dimensions in a computer game. Cogn. Emot. 18, 663–688 10.1080/02699930341000167

[B183] Van Reekum C. M. Scherer K. R. (1997). “Levels of processing in emotion-antecedent appraisal,” in Cognitive Science Perspectives on Personality and Emotion, ed. Matthews G. (Amsterdam: Elsevier Science B.V), 259–300

[B184] Voss K. E. Spangenberg E. R. Grohmann B. (2003). Measuring the hedonic of and utilitarian dimensions of consumer attitude. J. Mark. Res. 40, 310–320 10.1509/jmkr.40.3.310.19238

[B185] Watson D. Wiese D. Vaidya J. Tellegen A. (1999). The two general activation systems of affect: structural findings, evolutionary considerations, and psychobiological evidence. J. Pers. Soc. Psychol. 76, 820–838 10.1037/0022-3514.76.5.820

[B186] Wertheimer M. (1944). Gestalt theory. Soc. Res. (New York) 11, 78–99

[B187] Wierzbicka A. (1997). Understanding Cultures Through Their Key Words. New York: Oxford University Press

[B188] Wierzbicka A. (1999). Emotions Across Languages and Cultures: Diversity and Universals. Cambridge: Cambridge University Press

[B189] Wilson T. D. Centerbar D. B. Kermer D. A. Gilbert D. T. (2005). The pleasures of uncertainty: prolonging positive moods in ways people do not anticipate. J. Pers. Soc. Psychol. 88, 5–21 10.1037/0022-3514.88.1.5 15631571

[B190] Wilson T. D. Lindsey S. Schooler T. Y. (2000). A model of dual attitudes. Psychol. Rev. 107, 101–126 10.1037/0033-295X.107.1.101 10687404

[B191] Wolff P. H. (1987). The Development of Behavioral States and the Expression of Emotions in Early Infancy. Chicago: University of Chicago Press

[B192] Yik M. S. M. Russell J. A. Barrett L. F. (1999). Structure of self-reported current affect: I integration and beyond. J. Pers. Soc. Psychol. 77, 600–619 10.1037/0022-3514.77.3.600

[B193] Zeelenberg M. Pieters R. (2006). “Feeling is for doing: a pragmatic approach to the study of emotions in economic behavior,” in Social Psychology and Economics, eds De Cremer D. Zeelenberg M. Murnighan K. (Mahwah, NJ: Erlbaum), 117–137

[B194] Zeeman E. C. (1976). Catastrophe theory. Sci. Am. 4, 65–83 10.1038/scientificamerican0476-65

[B195] Zuckerman M. Kolin E. A. Price L. Zoob I. (1964). Development of a sensation-seeking scale. J. Consult. Psychol. 28, 477–482 10.1037/h0040995 14242306

